# Biochar for Electrochemical Sulfanilamide Detection: Comparative Evaluation of Carbon Paste and Screen-Printed Electrodes

**DOI:** 10.1021/acsomega.5c04276

**Published:** 2025-07-28

**Authors:** Lucas L. Cabral, Cristiane Kalinke, Marcia G. P. Valenga, Luiz H. Marcolino-Junior, Márcio F. Bergamini

**Affiliations:** † Laboratory of Electrochemical Sensors (LabSensE), Department of Chemistry, Federal University of Paraná, 81531-980 Curitiba, PR, Brazil; ‡ Institute of Advanced Materials (INAM), 16748 University Jaume I, Unnamed Road, 12006 Castelló de la Plana, Castelló, Spain

## Abstract

Antibiotics such as sulfanilamide (SFD) pose significant environmental and health risks due to their persistence in wastewater and their potential to contaminate food. However, conventional detection methods for these compounds are often costly and time-consuming. This study proposes activated biochar derived from sugar cane bagasse as a sustainable modifier for electrochemical sensors to detect SFD using adsorptive stripping voltammetry (AdSV). The biochar was functionalized with HNO_3_ (termed BCA) to enhance SFD preconcentration and was incorporated into two electrode platforms: carbon paste electrodes (CPEs) and screen-printed carbon electrodes (SPCEs). The preparation of each modified electrode (CPME-BCA and SPCE-BCA) was optimized to evaluate its analytical performance. Both BCA-modified electrodes exhibited significantly enhanced SFD oxidation signals compared to those of their unmodified counterparts. The optimized SPCE-BCA demonstrated a linear detection range from 5.0 × 10^–9^ to 5.0 × 10^–6^ mol L^–1^, a limit of detection of 1.5 × 10^–9^ mol L^–1^, and high reproducibility, with an RSD of 4.92%. The sensor was successfully applied to spiked samples of tap water, synthetic urine, and low-fat milk, achieving recoveries of between 90.7 and 111%. This work highlights sugar cane bagasse-derived biochar as a cost-effective and eco-friendly material for electrochemical sensing. Notably, SPCE-BCA required less biochar for modification than CPME-BCA, offering a scalable solution for SFD monitoring in diverse matrices.

## Graphical abstract

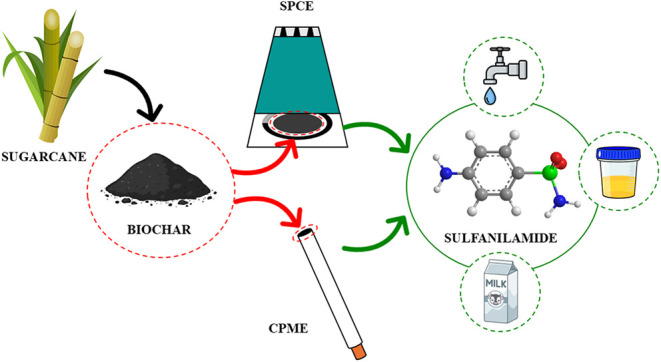

## 1 Introduction

Antibiotics are considered emerging contaminants,^1^ primarily found in aquatic environments due to excretion in biological fluids like urine, highlighting the inefficiency of conventional wastewater treatment.^2^ Moreover, improper antibiotic use can leave residues in food, such as milk, posing a risk to human health; these residues may trigger allergies, contribute to the development of resistant bacteria, and disrupt fermentation processes in dairy production.^3^ Consequently, monitoring compounds such as sulfanilamide (SFD) in environmental, biological, and food samples is essential. SFD is a synthetic antibiotic from the sulfonamide class, and it is known as the prominent degradation intermediate of different sulfonamide antibiotics.^4^ The antibacterial activity of SFD consists of competitively inhibiting the enzyme dihydropterase synthetase during the synthesis of folic acid, preventing the formation of bacterial nucleic acid, consequently suppressing its growth and proliferation.^5,6^

Traditional detection methods for SFD primarily rely on instrumental techniques, including chromatography,^7,8^ electrophoresis,^9,10^ and spectrophotometry.^11,12^ Generally, these methods are expensive and time-consuming, requiring a prior sample preparation step and involving many laboratory work steps.^13^ Compared to traditional methods, electrochemical techniques stand out due to their lower instrumental cost, fast response, high sensitivity, good selectivity, low detection limits, small sample volumes, and the possibility of on-site analysis.^4,14^

Despite the advantages of electroanalytical methods, graphite-based electrodes, such as carbon paste (CPE) and screen-printed carbon (SPCE), can present some performance problems.^15^ Therefore, modifying these electrodes by adding chemically active species aims to solve performance problems, either to obtain nonexistent electrochemical energy (in the case of in situ analyses) or to improve the preconcentration of compounds of interest (in the case of ex situ analyses).^16^ Moreover, depending on the modifier, it can improve the electron transfer and electroactive surface area of the electrodes, enhance stability, sensitivity, and refine the analytical signal.^15,17,18^ In this case, the modification using renewable materials is an interesting alternative, which provides an eco-friendly approach.^15^

Biochar is a carbon-rich product obtained through the thermal decomposition of organic matter when biomass is heated at moderate temperatures (<700 °C) and with a limited oxygen supply.^19,20^ The conversion of biomass into biochar has gained significant attention due to its economic advantages and potential for sustainable development.^21^ Biochar synthesis is considered eco-friendly, as biomass is often carbon-neutral; the carbon dioxide released during pyrolysis is effectively offset by the CO_2_ captured during photosynthesis.^22^ The biochar characteristics include a porous structure, high mineral content, and a surface with different functional groups, which are influenced by the types of pyrolysis (slow or fast), precursor biomass, and reactor where it is produced.^23,24^ Due to its characteristics, biochar has been applied as an adsorbent for pollutant removal,^23,25^ soil amendment,^26^ catalyst,^27^ fuel cells,^28^ and supercapacitors.^29^ The unique properties of biochar, such as its ability to interact with both organic and inorganic compounds aligned to sustainable environmental practices, make it a promising material for analytical and electrochemical applications, particularly in developing electrochemical sensors.^30–32^ Furthermore, their structural characteristics can contribute to analyte recognition and the transduction of this interaction as an electrical signal during redox reactions.^31^ The biochar has been successfully employed to modify various diverse electrode approaches (e.g., carbon paste, screen-printed carbon, and glassy carbon electrodes) to preconcentrate and detect metal ions,^33–36^ pesticides,^37–39^ phenolic compounds,^40,41^ biomarkers,^42,43^ among others.^30^ This is possible due to the versatility of biochar, which allows its application in composite materials, dispersions, microfluidic systems, anchoring of nanomaterials and biomolecules, (bio)­sensing, and energy, among others.^30^

Herein, we evaluated and compared different biochar processing strategies to construct modified electrodes, focusing on two approaches: (1) using a carbon paste-modified electrode (CPME) where biochar is incorporated in the bulk of the composite, altering the composition of the electrode; and (2) using a screen-printed carbon electrode (SPCE) where a dispersion of nanobiochar is applied via drop-casting, modifying the surface of the electrode. This dual approach provides a comprehension of how biochar processing methods impact electrode performance in the preconcentration of chemical species. For that, biochar was prepared from sugar cane bagasse, activated with HNO_3_ (50 vol %), characterized, and applied for sensing SFD in environmental, biological, and food samples. Unlike previously developed electroanalytical methods for SFD detection,^2,5,13,44–46^ biochar remains unexplored as an electrode modifier for this analyte preconcentration, highlighting the novelty in its application by providing an eco-friendly alternative for this approach.

## 2 Materials and Methods

### 2.1 Reagents and Materials

All reagents were analytical grade and used with no further purification. Graphite powder (Fischer Chemical), mineral oil (Merck), sulfanilamide ultrapure (>99%; Vetec, Brazil), acetic acid (99.8%; Neon), boric acid (99%; Carlo Erba), hydrochloric acid (36.5%; J. T. Baker), nitric acid (65%; Cinética), phosphoric acid (85%; Carlo Erba), and sodium hydroxide (>97%; Neon) were employed. The solutions used in this work were prepared using ultrapure water Milli-Q (Millipore) with a resistivity superior to 18.2 MΩ cm^–1^. The Britton–Robinson (B–R) buffer solution (0.1 mol L^–1^) was prepared by mixing 0.04 mol L^–1^ of acetic acid (CH_3_COOH), 0.03 mol L^–1^ of boric acid (H_3_BO_3_), and 0.03 mol L^–1^ of phosphoric acid (H_3_PO_4_). For pH adjustment, 0.1 mol of L^–1^ hydrochloric acid (HCl) and 0.1 mol of L^–1^ sodium hydroxide (NaOH) were used.

### 2.2 Biochar Preparation and Characterization

Sugar cane bagasse was obtained from commercial establishments in Curitiba city, Paraná state, Brazil. The preparation of biochar consisted of a preliminary stage of biomass treatment, drying the sugar cane bagasse in an oven at 100 °C for 48 h, followed by cutting and grinding using a knife mill (STAR FT 50, Fortinox). The particles with fractions lower than 80 mesh (0.177 mm) were separated after sieving for further stages. For biochar preparation, 10 g of biomass (<80 mesh) was allocated in a glass reactor for a tubular furnace (EDG FT-40), with the following pyrolysis conditions: a residence time of 60 min, a heating rate of 5 °C min^–1^, and a final temperature of 400 °C. All of the conditions were previously optimized.^33,35^ Afterward, the biochar was chemically modified by contact with nitric acid (HNO_3_) of 50 vol %.^19^ For this activation, a reflux system was set up using a round-bottom flask containing 50 mL of HNO_3_ and 1.0 g of biochar and maintained under constant stirring at 60 °C for 3 h. Then, the mixture was filtered, and the biochar was washed with distilled water until the pH became neutral. Subsequently, the material was dried in an oven at 100 °C for 24 h and stored for further steps.

The characterization of the sugar cane bagasse biochar (BC) and the activated biochar (BCA) was performed by scanning electron microscopy (SEM), energy-dispersive X-ray spectroscopy (EDS), Fourier transform infrared spectroscopy (FTIR), and water surface contact angle (θ). The photomicrographs to verify the surface morphology of the materials were obtained by using a scanning electron microscope (TESCAN VEGA3 LMU). The chemical composition of the materials was semiqualitatively determined by EDS using a system of chemical analysis (Oxford), coupled to a scanning electron microscope. The functional groups were detected by FTIR using a spectrophotometer (α II, Bruker) in the range from 4000 to 400 cm^–1^, and a scan accumulation of 32 scans. The wettability of the electrodes was measured by the contact angle between a drop of distilled water (2.0 μL) and the working electrode surface. A Nova digital microscope camera captured the images at room temperature, and the angle analysis was performed using the free software ImageJ (USA) with the contact angle plugin tool. Analysis of variance (ANOVA) and Tukey test were employed using the software Statistica (version 10.0, during the free trial license) to determine the possible differences in the wettability of each electrode.

### 2.3 Electrode Construction

This study evaluated the performance of two different electrodes: the carbon paste electrode (CPE) and the screen-printed carbon electrode (SPCE). In both platforms, to verify the analytical response promoted with the biochar modification, biochar was used without activation (CPME-BC and SPCE-BC) and activated with 50 vol % of HNO_3_ (CPME-BCA and SPCE-BCA). Figure 1 presents a scheme for the electrode’s preparation.

**Figure 1:**
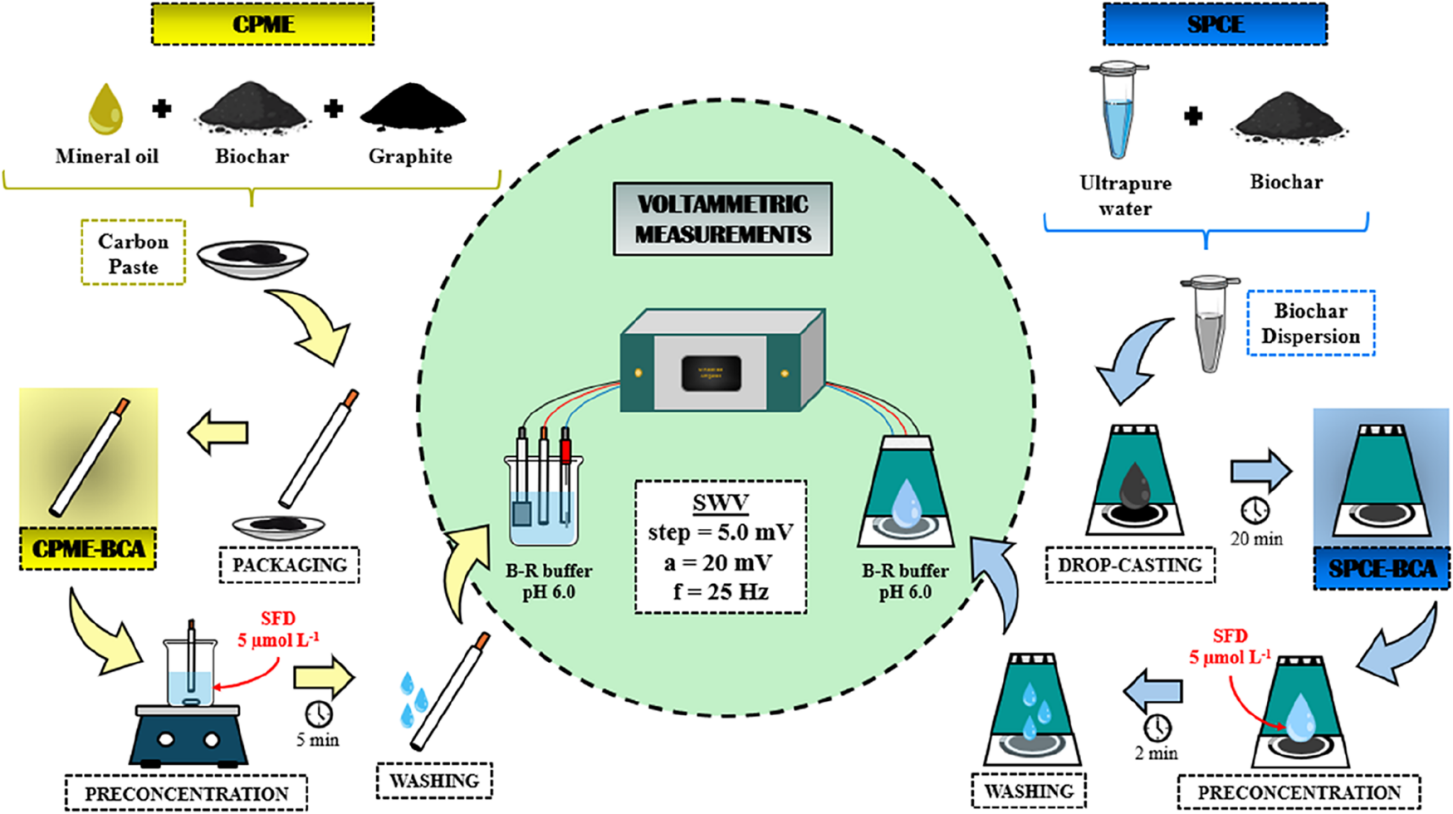
Scheme for the electrode modification and voltammetric measurements: CPME (left) and SPCE (right).

For the CPME, the carbon paste was prepared by mixing mineral oil (25 wt %) as the binder, graphite powder (50 wt %) as the conductive material, and biochar (25 wt %) as the modifier. The carbon paste was inserted into electrode supports constituted by a PVC tube with an internal diameter of 3.0 mm and a copper wire inside it, which was responsible for the electrical contact of the electrode. For the SPCE, a biochar dispersion was drop-casted on the surface of a commercial SPCE (Metrohm Dropsens 110) formed by carbon working and counter electrodes and a silver ink pseudoreference electrode. The SPCE modification procedure proposed by Valenga et al.^42^ was adopted. It consists of homogenizing the biochar for 15 min using a pestle and mortar and preparing a biochar dispersion (*v* = 1.0 mL) in ultrapure water at a proportion of 1.0 mg mL^–1^ using an ultrasonic bath for 30 min. Then, the dispersion was centrifuged at 7500 rpm for 2 min, the supernatant was collected, and the dispersion was acidified using 1.0 mol L^–1^ of HCl until the final acid concentration was 0.1%. By the difference between the initial mass of the weighed biochar and the remaining mass after collecting the supernatant, the estimated final biochar concentration in the dispersion used for drop-casting is approximately 0.1 mg mL^–1^. For the modification, 5.0 μL of biochar dispersion was dripped onto the surface of the working electrode and then dried for 20 min at 60 °C until complete evaporation of the solvent.

### 2.4 Voltammetric Measurements

The voltammetric measurements were performed in a potentiostat/galvanostat (μAutolab Type III) by using the software NOVA 1.10.4 for data treatment. For the evaluation of both electrodes, the square wave voltammetry (SWV) technique was used for the detection of SFD in 0.1 mol L^–1^ of B–R buffer as the supporting electrolyte in a potential range from +0.6 to +1.6 V (vs. Ag|AgCl for the CPME and Ag for the SPCE), with a step potential of 5.0 mV, a wave amplitude of 20 mV, and a frequency of 25 Hz. Parameters related to the experimental conditions for the SFD determination using the CPME-BCA (i.e., biochar proportion, preconcentration pH and time, and measurement solution pH) and the SPCE-BCA (i.e., biochar proportion and preconcentration time) were previously evaluated and optimized, as shown in Table S1. To evaluate the possible differences in voltammetric responses of each electrode, ANOVA and Tukey test were performed using the software Statistica (version 10.0) during the free trial license.

Regarding the CPME, a set of three electrodes were employed: a working electrode (CPE or CPME), a platinum counter electrode, and a reference electrode (Ag|AgCl|3.0 mol L^–1^ KCl). The working electrode was immersed in a solution containing 5.0 μmol L^–1^ of SFD in B–R buffer pH 2.0 for 2 min under constant stirring to promote the preconcentration of the analyte under open-circuit potential condition. Then, it was gently washed with distilled water to remove the excess of SFD molecules that had not interacted with the biochar on the electrode surface. Subsequently, the working electrode was transferred to another cell for voltammetric measurement in B–R buffer pH 6.0 to determine SFD. After the measurements were performed using the CPME, a cleaning procedure by cyclic voltammetry (5 cycles) was adopted in the potential range of +0.4 to +1.6 V and a scan rate of 100 mV s^–1^ to remove the analyte molecules from the electrode surface (Figure S1). For the SPCE, 10 μL of a solution containing 5.0 μmol L^–1^ of SFD was dripped onto the surface of the working electrode for the analyte preconcentration step for 2 min at open circuit. Then, the electrode surface was washed by immersion in a flask containing distilled water for 3 s. Afterward, 80 μL of the supporting electrolyte (B–R buffer, pH 6.0) was dropped onto the electrode surface for the voltammetric detection of SFD. In this case, after the measurements, the SCPE disposable electrode was replaced with a new electrode. Following the optimization of experimental parameters, the analytical performance of the sensors was characterized by determining the linear dynamic range, sensitivity, limit of detection (LOD = 3 SD/slope), and limit of quantification (LOQ = 10 SD/slope).^41,47^

### 2.5 Evaluation of Concomitant Species and Determination of Sulfanilamide in Tap Water, Synthetic Human Urine, and Low-Fat Milk Samples

To validate the proposed method, the SPCE-BCA was employed to determine SFD in tap water, synthetic human urine, and low-fat milk samples. Common compounds found in the studied samples were investigated as concomitant species, such as ascorbic acid (AA), citric acid (CA), calcium ions (Ca^2+^), casein (CAS), glucose (GLU), lactose (LAC), sodium ions (Na^+^), sulfamethoxazole (SMX), and urea (UR). The influence of these species was evaluated at three concentration levels (0.50, 5.0, and 50 μmol L^–1^) in the presence of a 5.0 μmol L^–1^ SFD.

The determination was performed in tap water, synthetic urine, and milk-spiked samples. The tap water sample was collected from the Department of Chemistry at the Federal University of Paraná and stored at 4 °C in a sterilized plastic bottle until use. The synthetic human urine sample was prepared according to Baccarin et al.^48^ by mixing 25.0 g of urea (C_2_H_4_N_2_O), 1.10 g of calcium chloride dihydrate (CaCl_2_·2H_2_O), 2.92 g of sodium chloride (NaCl), 1.60 g of potassium chloride (KCl), 2.25 g of sodium sulfate (Na_2_SO_4_), 1.00 g of ammonium chloride (NH_4_Cl), and 1.40 g of potassium phosphate monobasic (KH_2_PO_4_), dissolved in ultrapure water (*v* = 1.0 L). The low-fat milk sample was commercially acquired in a local supermarket in Curitiba city, Paraná state, Brazil. For the SFD determination, the samples were spiked at different levels of the SFD standard solution: 10, 30, and 50 μmol L^–1^ for water and urine and 100, 300, and 500 μmol L^–1^ for milk. The spiked samples were diluted in 0.1 mol L^–1^ of B–R buffer solution (pH 2.0) in a ratio of 1:10 for water and urine and 1:100 for milk, reaching a final concentration of 1.0, 3.0, and 5.0 μmol L^–1^ of SFD for all samples. The measurements were performed by an SWV-optimized technique.

## 3 Results and Discussion

### 3.1 Characterization of the Sugar Cane Bagasse Biochar

The surface morphology of pristine biochar (BC; Figure S2A) revealed a heterogeneous structure characterized by irregular cavities, overlapping plate-like features (likely remnants of lignocellulosic biomass), and a mix of smooth and rough regions. In contrast, nitric acid-activated biochar (BCA; Figure 2A) retained structural heterogeneity but displayed increased surface fragmentation, with a higher density of cavities and the absence of plate-like structures. These structural characteristics suggest an increased availability of adsorption sites for SFD molecules. These changes may have occurred due to the contact of the biochar with nitric acid during the activation, promoting higher wear of the biochar surface due to its oxidizing and erosive character.^19^ Figure 2B shows BCA particles after drop-casting onto the electrode surface, revealing a uniform distribution and homogeneous surface coverage. This uniformity is critical for achieving reproducible sensor performance during fabrication. Additionally, BCA exhibited irregular, angular granules with reduced particle sizes (ranging from microscale in BC to nanoscale in BCA), which may further contribute to improved electrochemical activity by increasing the effective surface area. Carbon nanomaterials are well-known for enhancing surface area,^18^ which also probably occurred in the modification of SPCE, since there is a decrease in BCA particle size in the dispersion to the nanoscale, as suggested by SEM images.

**Figure 2:**
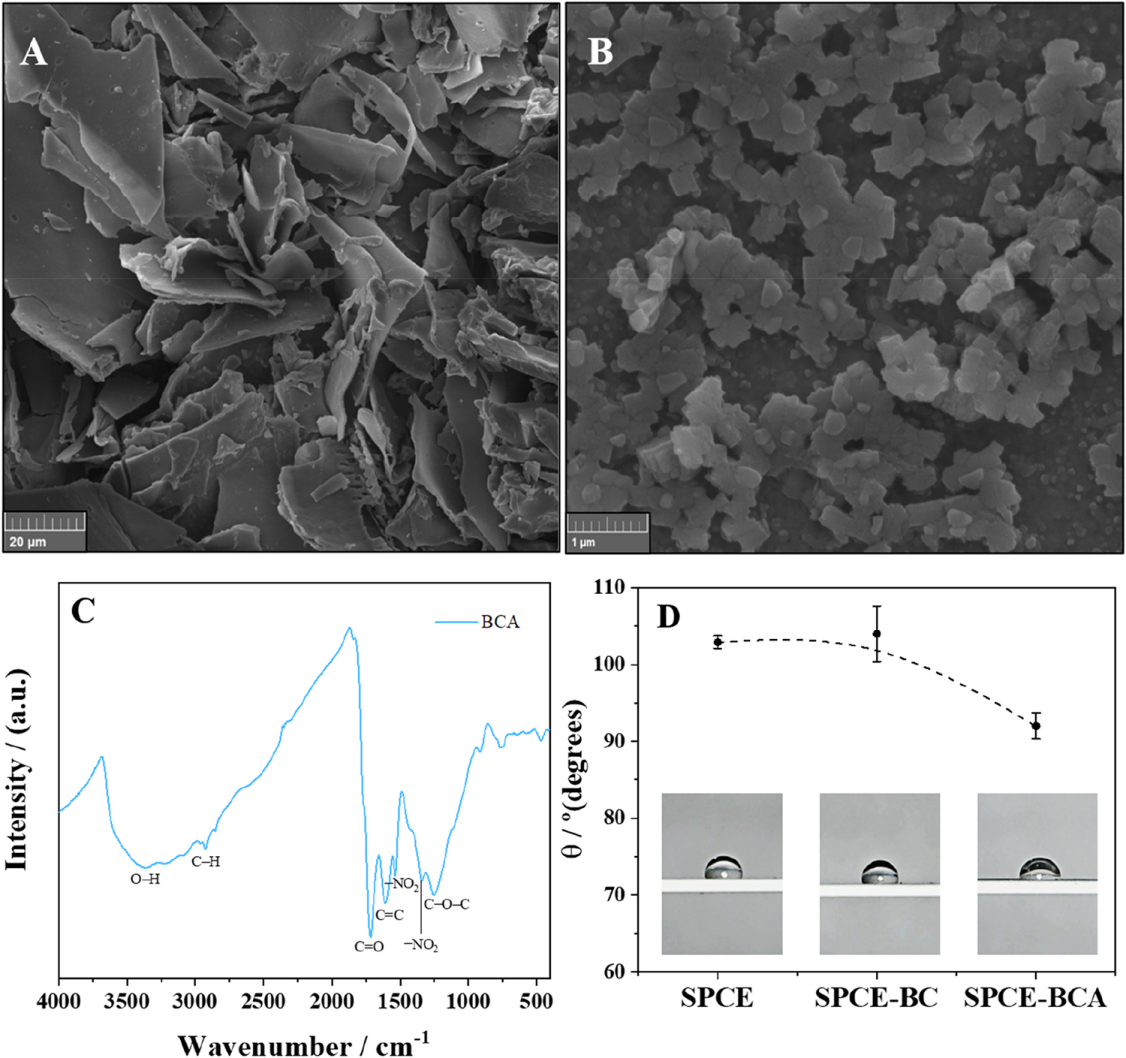
(A) SEM photomicrographs for BCA at a magnification of 2000×, (B) SEM photomicrographs for BCA dispersion at a magnification of 50,000×, (C) FTIR spectrum for BCA, and (D) contact angle for screen-printed carbon electrodes.

The EDS spectra for BC and BCA (Figure S2B) showed that the semiquantitative composition of the materials is similar, with the presence of the elements carbon (C), oxygen (O), silicon (Si), magnesium (Mg), phosphorus (P), potassium (K), and calcium (Ca). These elements are commonly found in biochar from different biomasses such as castor oil cake,^19^ maize straw,^49^ and rice straw.^50^ It is essential to highlight that even for BCA, the presence of Si, Mg, P, K, and Ca indicates that activation with HNO_3_ did not remove these elements from BC, suggesting the possible presence of these minerals in the form of carbonates, oxides, silicates, and phosphates in the activated biochar.^51^

The FTIR spectra for BC (Figure S2C) and BCA (Figure 2C) show similar bands. A broad band at around 3400 cm^–1^ indicates O–H stretching, suggesting the presence of phenolic and/or carboxylic groups.^23^ The band at around 2930 cm^–1^ is due to aliphatic C–H stretching.^33^ At 1710 cm^–1^, there is a characteristic band of the C=O stretching vibration of the carboxylic group.^52^ At around 1615 cm^–1^, the presence of C=C stretching vibration of aromatic rings^19^ was observed. The bands in the range from 1400 to 1200 cm^–1^ are related to various functional groups such as −OH of phenols and carboxylic acids^33^ and C–O–C of alkyl aromatics,^53^ which leads to overlapping bands. While the FTIR spectra of BC and BCA exhibit similarities, some differences were noted. The bands at 1710 and 1250 cm^–1^, corresponding to functional groups such as carboxylic acids, phenols, and ketones, were more intense in the BCA spectrum than those in BC. This increase in intensity indicates a higher degree of oxidation in the carbonaceous structure^33^ of BCA compared to that of BC. The oxidative modification of biochar by nitric acid predominantly targets aliphatic chains, while aromatic rings remain stable and unaltered. Oxidation of single-carbon aliphatic chains results in the formation of ketone groups, whereas the oxidation of aliphatic chains tends to form dicarboxylic groups.^33,54,55^ Furthermore, the appearance of bands at 1530 and 1340 cm^–1^ in the BCA spectrum is attributed to the presence of −NO_2_ groups, formed as a result of nitration reactions occurring concomitantly with the oxidative processes^33,54^ during the BC activation step.

For carbon paste electrodes, the water contact angle (θ) measurements revealed an average of 67.1 ± 0.2° for CPE, 106 ± 1° for CPME-BC, and 98.9 ± 1.3° for CPME-BCA (Figure S2D). CPE presented a hydrophilic characteristic, while CPME-BC and CPME-BCA presented a hydrophobic characteristic,^56^ suggesting that biochar influences the electrode surface characteristics. To statistically confirm this difference, ANOVA and the Tukey test were performed (Table S2). ANOVA revealed a significant difference in the contact angles for carbon paste electrodes since *F* calculated was higher than *F* critical (*F*_calc_ > *F*_crit_; 739.6 > 9.55). Using the Tukey test, comparison of the carbon paste electrodes revealed that the contact angles for the electrodes studied significantly differ. This result indicates that the modifier contributes to the hydrophobic character of electrodes; however, between CPME-BC and CPME-BCA, the activation with HNO_3_, which inserted polar functional groups at the biochar surface, diminishes the hydrophobic characteristic of this electrode. Thus, nonpolar π–π interactions could favor the preconcentration of SFD on the biochar.

The water contact angle measurements (Figure 2D) revealed average values of 103 ± 1° for bare SPCE, 104 ± 4° for SPCE-BC, and 92.0 ± 1.7° for SPCE-BCA. All surfaces remained hydrophobic (θ > 90°), with SPCE-BC displaying the highest hydrophobicity and SPCE-BCA the lowest. The reduced hydrophobicity of SPCE-BCA likely stems from HNO_3_ activation, which introduces polar functional groups (e.g., −COOH, −OH) onto the biochar surface, enhancing its affinity for water. This shift toward hydrophilicity aligns with the increased availability of adsorption sites for polar sulfanilamide molecules, improving the sensor performance. Furthermore, the existence of more hydrophilic groups in BCA favors the interaction between biochar and sulfanilamide, leading, for example, to the formation of hydrogen bonds,^57^ which may be advantageous for the preconcentration and detection of this analyte. Analysis of variance and the Tukey test were performed to statistically confirm this differentiation of the SPCE surface with respect to the water contact angle (Table S2). The ANOVA results showed that the contact angles significantly differ between the electrodes, given that the calculated *F* was higher than the critical *F* (*F*_calc_ > *F*_crit_; 15.82 > 9.55). The Tukey test showed that the contact angle average values for SPCE and SPCE-BC are statistically equal; i.e., both have the same hydrophobic character of their surfaces.

Despite using the same modifier (BCA), SEM images revealed distinct morphological differences between its powdered form (used in CPE modification) and its dispersed state (applied to SPCE). These structural variations likely impart unique electrochemical properties to each sensor, contributing to their divergent performances. Contact angle measurements corroborate those different characteristics: in CPME-BCA, BCA modification rendered the sensor more hydrophobic than the bare CPE, whereas in SPCE-BCA, BCA decreased the hydrophobicity relative to the bare SPCE. The more pronounced change in wettability for CPME-BCA (vs. SPCE-BCA) may arise from the higher BCA content incorporated into the carbon paste matrix during modification. This suggests that the greater surface coverage of BCA in CPME-BCA amplifies its inherent hydrophobic character, while the lower BCA loading in SPCE-BCA allows the polar functional groups introduced by HNO_3_ activation to dominate, enhancing the hydrophilicity. These findings highlight how modifier loading and electrode platform architecture synergistically govern surface wettability—a critical factor controlling analyte–sensor interactions in aqueous-based samples such as SFD.

### 3.2 Electrochemical Behavior of CPME-BCA and SPCE-BCA

The SWV technique was employed to verify the peak current influence promoted by the presence of biochar in the modified electrodes due to its capacity for preconcentrated chemical species. The results presented in Figure 3A,C demonstrate the characteristic voltammetric profile of SFD oxidation obtained for CPEs and SPCEs, respectively. The oxidation process occurs in the phenylamine group in the SFD structure, resulting in a free-radical formation.^2,45,46^ Posteriorly, two free radicals rapidly combine in a dimerization reaction, forming the hydrazobenzenesulfonamide.^46^ The redox process of SFD is presented in Figure S3.

**Figure 3:**
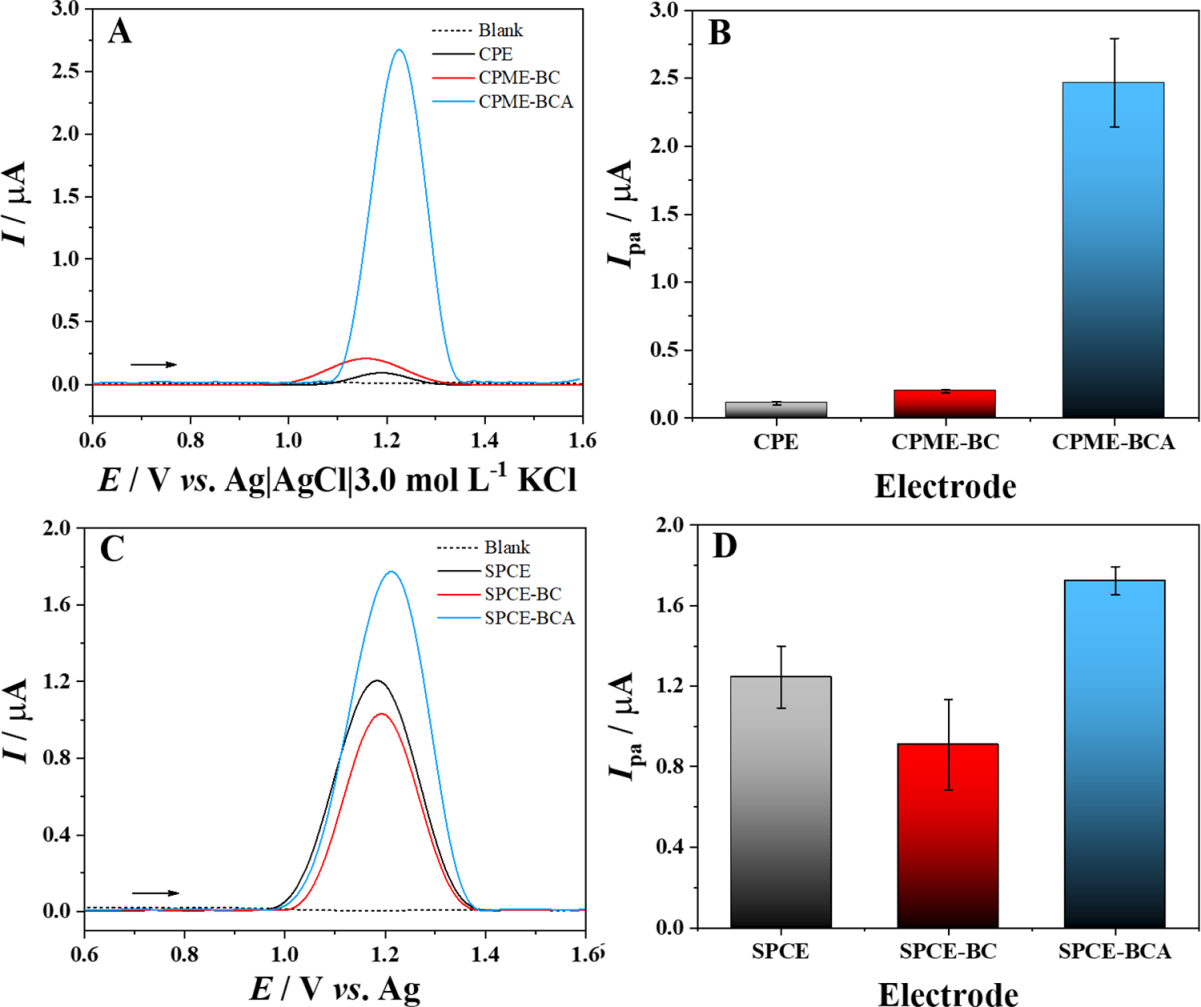
SWV response and anodic peak currents obtained for 5.0 μmol L^–1^ of SFD detection using CPME-BCA (A, B) and SPCE-BCA (C, D) electrodes. Supporting electrolyte: B–R buffer, pH 6.0.

For carbon paste electrodes (CPE, CPME-BC, and CPME-BCA), lower anodic peak current (*I*_pa_) values were obtained for CPE and CPME-BC, resulting in 0.11 ± 0.01 and 0.12 ± 0.01 μA, respectively, as shown in Figure 3B. Conversely, for the CPME-BCA, an increase of the anodic peak current was observed (*I*_pa_ = 2.5 ± 0.3 μA), suggesting that the activated biochar has contributed to the improvement in the SFD preconcentration and oxidation signal. The ANOVA test statistically confirmed this contribution of BCA in the increase of SFD oxidation signal, showing a significant difference between the three electrodes since *F*_calc_ > *F*_crit_ (151 > 5.14), as shown in Table S3. Then, the Tukey test showed no significant difference between the response signals obtained for CPE and CPME-BC. At the same time, a significant difference between the signals for CPME-BCA and CPE and between CPME-BCA and CPME-BC was observed. Therefore, it is possible to state that the activated biochar present in the carbon paste contributed substantially to the increase in the response signal for SFD determination.

The screen-printed carbon electrodes (SPCE, SPCE-BC, and SPCE-BCA) also had differences, although they were less pronounced than those for the carbon paste electrodes. It is possible to notice that the Ipa values for SPCE, SPCE-BC, and SPCE-BCA were 1.2 ± 0.2, 0.91 ± 0.22, and 1.7 ± 0.1 μA, respectively (Figure 3D). Significative differences between the screen-printed carbon electrodes were pointed out by ANOVA (Table S3), in which the calculated F value resulted in a superior value compared to the critical *F* value (*F*_calc_ > *F*_crit_; 19.0 > 5.14). According to the Tukey test, the SPCE and SPCE-BC peak current values are statistically equal. At the same time, SPCE-BCA significantly differs from SPCE and SPCE-BC peak current values for SFD oxidation.

The increase in the response signal for both electrodes modified with activated biochar (CPME-BCA and SPCE-BCA) is possibly related to an improvement in the preconcentration of SFD on the electrode surface due to the interaction between the analyte and the modifier. The FTIR analysis confirmed the presence of oxygenated functional groups, such as carbonyl (C=O), hydroxyl (O–H), nitro (−NO_2_), and carboxyl (−COOH) groups, on the BCA surface. These groups contribute to hydrogen-bonding interactions between oxygen and nitrogen atoms of biochar and the hydrogen atoms in the aromatic ring and amine groups of the SFD structure. Furthermore, the SFD molecule contains oxygen and nitrogen atoms, which can form hydrogen bonds^13^ with the hydrogen atoms of the BCA groups. This mutual availability of hydrogen bond donors and acceptors enhances the probability of hydrogen-bonding interactions between the BCA surface and the SFD molecules. In addition, another possible mechanism may be the π–π stacking interactions between the aromatic ring of the phenylamine group of SFD and the aromatic structures^38,57^ on the biochar surface, contributing to the adsorption process. Figure S4 presents a scheme of the proposed interactions between the BCA surface and the SFD molecules.

Although there was an improvement in the response signal for both sensors modified with BCA, the peak current intensity differed between them. The stronger signal observed for CPME-BCA (25 wt %) compared to that for SPCE-BCA (0.1 mg mL^–1^ dispersion) is attributed to the higher amount of modifier on the electrode surface, offering more adsorption sites for SFD accumulation. In contrast, SPCE-BCA’s thinner BCA film provides fewer available adsorption sites, resulting in a lower peak current. To assess whether the surface area contributes to this behavior, the electrochemically active area of unmodified (SPCE, CPE) and modified electrodes (SPCE-BC, SPCE-BCA, CPME-BC, CPME-BCA) was estimated in 0.1 mol L^–1^ of KCl containing 5 mM [Fe­(CN)_6_]^3‑/4‑^, using the Randles–Ševčík equation applied to the peak currents at scan rates ranging from 5 to 100 mV s^–1^ (*n* = 3).^58^ For carbon paste platforms, the area values were CPE = 0.020 ± 0.001_7_ cm^2^, CPME-BC = 0.0178 ± 0.0006_0_ cm^2^, and CPME-BCA = 0.030 ± 0.009_9_ cm^2^. For screen-printed platforms: SPCE = 0.085 ± 0.006_1_ cm^2^, SPCE-BC = 0.095 ± 0.009_3_ cm^2^, and SPCE-BCA = 0.079 ± 0.005_7_ cm^2^. One-way ANOVA (*p* > 0.05) revealed no statistically significant differences in electrochemically active area between modified and unmodified electrodes for either platform. These results suggest that the observed enhancement in sensitivity toward sulfanilamide detection primarily stems from the adsorptive properties of the biochar modifiers rather than from changes in the electrochemically active area. The sorption properties of biochar have expanded its electrochemical applications;^30^ moreover, other studies have also reported its successful use as an electrode modifier for the determination of various pharmaceuticals.^59–63^ These studies highlight the role of biochar as an effective modifier in the electrochemical determination of pharmaceuticals, enhancing response signals and detectability.

### 3.3 Analytical Performance of CPME-BCA and SPCE-BCA

The experimental parameters for SFD determination were evaluated to provide better voltammetric responses by using both electrodes. For the CPME-BCA, the optimized conditions were 25 wt % of biochar proportion in the carbon paste, preconcentration in B–R buffer pH 2.0 for 5 min, and measurements performed in B–R buffer pH 6.0. For the SPCE-BCA, the proportion of biochar of 0.1 mg mL^–1^ in the dispersion (after centrifugation and supernatant separation), preconcentration in B–R buffer pH 2.0 for 2 min, and measurements performed in B–R buffer pH 6.0. Figures S5 and S6 and Table S1 show the results obtained for the experimental optimization, which is described in the Supporting Information. The analytical performances of both CPME-BCA and SPCE-BCA electrodes were evaluated for the detection of SFD, and the results are shown in Figure 4. For the CPME-BCA, the analytical curve was obtained in the linear range (*R* ^2^ = 0.990) from 5.0 × 10^–6^ to 1.0 × 10^–4^ mol L^–1^ SFD (Figure 4A), with a sensitivity of 0.24 μA L μmol^–1^, following the equation *I*_pa_ (μA) = 0.842 + 0.239 C_SFD_ (μmol L^–1^). The calculation for the limits of detection and quantification (LOD and LOQ) resulted in 0.13 and 0.40 μmol of L^–1^, respectively. A reproducibility study for CPME-BCA showed promising results (RSD = 7.01%) for 10 different electrodes (intraday) for the detection of 50 μmol L^–1^ of SFD (Figure 4B). In addition, the physical characteristics of the carbon paste significantly changed after approximately 100 days, acquiring a drier consistency and making electrode construction more difficult, which also led to a decrease in the SFD response signal. Therefore, the carbon paste was considered to be stable only for this period (100 days) when stored in a hermetically sealed plastic flask.

**Figure 4:**
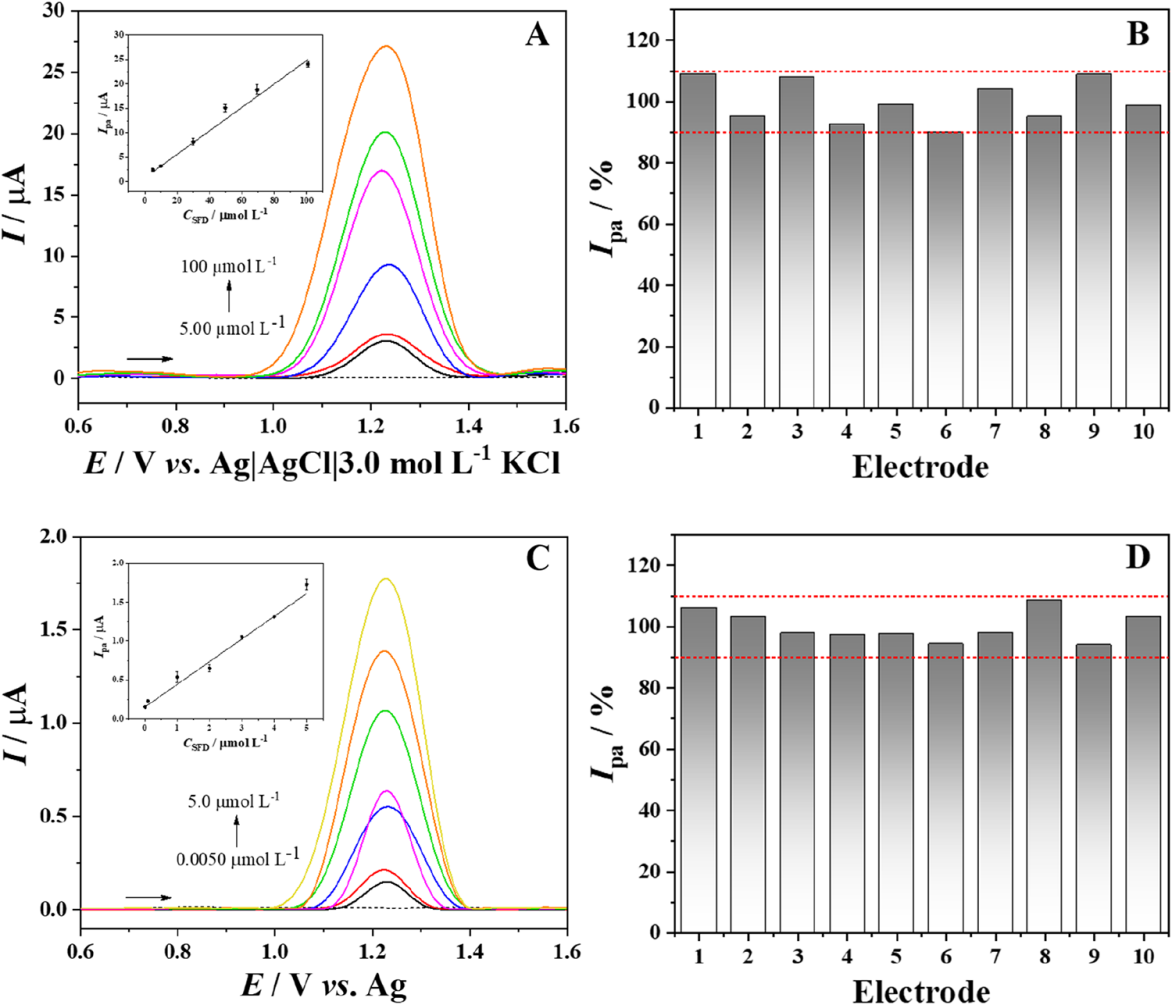
Voltammetric responses and analytical curves obtained for (A) CPME-BCA (5.00–100 μmol L^–1^ SFD) and (C) SPCE-BCA (0.0050–5.0 μmol L^–1^ SFD), with respective precision (intraday) for 10 different electrodes for (B) CPME-BCA and (D) SPCE-BCA. Supporting electrolyte: B–R buffer, pH 6.0.

Regarding the SPCE-BCA, a linear range (*R* ^2^ = 0.998) from 5.0 × 10^–9^ mol L^–1^ to 5.0 × 10^–6^ mol L^–1^ SFD was achieved (Figure 4C), showing a similar sensitivity of 0.29 μA L μmol^–1^, following the equation *I*_pa_ (μA) = 0.153 + 0.291 C_SFD_ (μmol L^–1^). The limits of detection and quantification calculated were 1.5 and 4.9 nmol L^–1^, respectively. In addition, better reproducibility was obtained for the SPCE-BCA (RSD = 4.92%), applied for 10 different electrodes for the detection of 5.0 μmol L^–1^ of SFD (Figure 4D). Differences observed in the LODs can be attributed to the standard deviation of the blank measurements, which was significantly higher for CPME-BCA (4.46 × 10^–3^ μA) compared to that for SPCE-BCA (1.42 × 10^–4^ μA). This suggests that the drop-casting method used for modifying the SPCE provides a more uniform and consistent surface, whereas the preparation of the composite electrode (CPME) may lead to slight variations in the homogeneity of the electrode material across multiple surfaces. These variations increase the baseline noise and consequently increase the LOD for the CPME-BCA sensor.

The analytical performance of the proposed sensors was compared to that of other electroanalytical methods developed for the determination of SFD, as presented in Table 1. In this study, it is essential to highlight that the modification of the electrodes using activated biochar combines the cost-effectiveness of the modification step with the waste material, resulting in a feasible, low-cost, fast, simple, and eco-friendly procedure.^30,64^ In addition, the good performance achieved by both sensors is attributed to the adsorptive ability of BCA, with the spontaneous preconcentration of the SFD on the electrode surface. Furthermore, the SPCE-BCA sensor showed better electrochemical performance than the CPME-BCA and to other reported studies for the detection of SFD, highlighting its wide linear range and high sensitivity.

**Table 1:** Comparative Analytical Performance of Electrochemical Methods for SFD Determination^a^

electrode	technique	LDR/μmol L^–1^	LOD/μmol L^–1^	refs
3DP CB/PLA	C-SWV	1.99 – 10.8	0.2	^2^
	SWV	2.99 – 8.86	1.2	
SiG-SPE	SWV	2.00 – 20.0	0.34	^44^
3DP CB/PLA	SWV	1.00 – 10.0	0.26	^45^
Ag@Pt–Rh NC	DPV	2.60 – 320	0.26	^4^
CoPc/CT/GCE	DPV	1.00 – 53.0	0.27	^5^
N–Cu-MOF	DPV	0.01 – 58.3	0.003	^65^
GCE-ND	SWV	1.20 – 581	0.94	^66^
GCE	SWV	3.00 – 250	0.64	^67^
AuNPs/Gr/GCE	DPV	0.10 – 1000	0.011	^68^
CPME-BCA	SWV	5.00 – 100	0.13	this study
SPCE-BCA		0.0050 – 5.0	0.0015	

a 3DP CB/PLA, 3D printed carbon black/polylactic acid electrochemically activated electrode; SiG-SPE, silica-graphite stencil printed electrode; Ag@Pt–Rh NC, glassy carbon electrode modified with Ag@Pt–Rh core–shell nanocubes; CoPc/GT/GCE, glassy carbon electrode modified with chitosan and cobalt phthalocyanine composite; N–Cu-MOF, glassy carbon electrode modified with nitrogen-doped copper-based metal–organic framework; GCE-ND, glassy carbon electrode modified with nanodiamond; GCE, glassy carbon electrode; AuNPs/Gr/GCE, glassy carbon electrode modified with Au nanoparticle-functionalized graphene; CPME-BCA, carbon paste electrode modified with biochar activated with HNO_3_; SPCE-BCA, screen-printed carbon electrode modified with biochar activated with HNO_3_; C-SWV, cyclic square wave voltammetry; SWV, square wave voltammetry; DPV, differential pulse voltammetry.

The superior analytical performance of SPCE-BCA over CPME-BCA, along with its enhanced reproducibility, can be attributed to the nanoscale particle size and increased surface area of the biochar. The drop-casting method used for SPCE-BCA modification produced biochar particles in the nanoscale range (Figure 2B), which exhibit a significantly larger specific surface area compared with the microscale particles in CPME-BCA (Figure 2A). This nanoscale morphology increases the density of adsorption sites available for SFD preconcentration, enabling efficient analyte accumulation even at low concentrations. Additionally, modifier loading and distribution significantly influence performance. While CPME-BCA contains a higher mass fraction of biochar (25% in carbon paste), its microscale particles are unevenly distributed within the porous carbon paste matrix, limiting accessible adsorption sites. In contrast, SPCE-BCA uses a smaller quantity of biochar (0.1 mg mL^–1^) but ensures a uniform dispersion and surface coverage via drop-casting. This optimizes the exposure of nanoscale BCA particles to the analyte, amplifying sensitivity and enabling detection at trace levels.

Notably, the CPME-BCA exhibits higher sensitivity compared to the SPCE-BCA, despite differences in electrode construction. This behavior can be explained by the higher biochar loading in the CPE, which increases the density of active sites available for SFD adsorption, leading to enhanced preconcentration and thus higher sensitivity. In contrast, the SPE modification involves a thinner biochar film, which provides better electron-transfer kinetics but less surface coverage, resulting in a comparatively lower sensitivity. Thus, the higher sensitivity of the CPME-BCA sensor arises primarily from its increased biochar loading and adsorption capacity, while the SPCE-BCA sensor balances sensitivity with improved electron transfer and lower background noise. Because the biochar layers are porous and irregular, normalizing the current by geometric area (current density) would not accurately reflect the effective surface or analyte interactions. Therefore, absolute current values were used to better represent the actual sensor performance.

### 3.4 Effect of Concomitant Species and Application of SPCE-BCA in Real Samples for Sulfanilamide Determination

Due to several functional groups on its surface, the biochar can interact and adsorb several organic and inorganic species. Therefore, competition may occur between the analyte and these species (when present in the same matrix) for the adsorptive sites of the biochar, especially during the preconcentration stage, interfering with the peak current signal and suppressing or increasing the analytical response of SFD. For this study, ascorbic acid (AA), citric acid (CA), casein (CAS), glucose (GLU), calcium ions (Ca^2+^), sodium ions (Na^+^), lactose (LAC), sulfamethoxazole (SMX), and urea (UR) were evaluated at three concentration levels (0.5, 5.0, and 50 μmol L^–1^), aiming the detection of 5.0 μmol L^–1^ of SFD. The absence of relevant influences on the response signal of SFD in the presence of the concomitant species suggests the good selectivity of the sensor, as shown in Figure S7. The obtained relative standard deviations (<7.55%) demonstrate the high precision of the measurements using the SPCE-BCA and indicate that there are no significant interferences (Table S4).

Thus, the determination of SFD was performed in tap water, synthetic human urine, and low-fat milk-spiked samples at 1.0–5.0 μmol L^–1^. The results showed success in quantifying the SFD in all samples based on the recovered values, as shown in Table 2. For the tap water sample, the recovery ranged from 98.5 to 101%; for the synthetic urine sample, from 98.7 to 111%; and for the low-fat milk sample, from 90.7 to 104%. Therefore, these values demonstrate that the developed sensor and the optimized method are interesting alternatives for monitoring low concentrations of sulfanilamide in different samples.

**Table 2:** Quantification of SFD in Tap Water, Synthetic Human Urine, and Low-Fat Milk Samples Using the SPCE-BCA Sensor

sample	added/μmol L^–1^	found/μmol L^–1^	recovery/%
water	1.00	1.01 ± 0.01	101
	3.00	2.95 ± 0.02	98.5
	5.00	4.98 ± 0.41	99.5
urine	1.00	1.11 ± 0.03	111
	3.00	2.97 ± 0.23	99.1
	5.00	4.93 ± 0.50	98.7
milk	1.00	1.04 ± 0.06	104
	3.00	2.77 ± 0.07	92.2
	5.00	4.54 ± 0.12	90.7

Detecting low concentrations of SFD in environmental, biological, and food samples is essential due to its persistence, potential to disrupt ecosystems, and contribution to the development of antibiotic-resistant bacteria. Residual SFD in water and food products poses health risks, including allergic reactions and diminished antibiotic effectiveness. Therefore, highly sensitive and selective analytical methods are required to meet regulatory standards and ensure public safety. Furthermore, early detection facilitates preventive monitoring and timely remediation, minimizing the risk of contamination and supporting effective environmental and food quality control.

## 4 Conclusions

This study demonstrated the effective use of sugar cane bagasse-derived biochar as a sustainable modifier to enhance the electrochemical detection of sulfanilamide (SFD) across two electrode platforms. Functionalization with nitric acid endowed the biochar (BCA) with a heterogeneous surface morphology and oxygenated functional groups, which facilitated strong interactions with SFD during preconcentration, significantly improving the sensor responses. Both carbon paste (CPME-BCA) and screen-printed electrodes (SPCE-BCA) modified with BCA exhibited better responses compared to their respective unmodified electrodes. However, the SPCE-BCA platform exhibited superior sensitivity and a broader operational range, enabling reliable trace-level SFD detection. This enhanced performance was explained by the nanoscale structuring and uniform dispersion of the biochar modifier on SPCE-BCA, which optimizes analyte accessibility compared with the microscale, unevenly distributed biochar within the carbon paste matrix. The sensor’s practical applicability was confirmed by successful testing in complex matrices (tap water, synthetic urine, and dairy products), where it demonstrated robust recovery and high selectivity against interfering species. By converting agricultural waste into a high-performance sensing material, this work highlights the potential of biochar-based modifiers to advance eco-friendly electrochemical platforms. These findings underscore the critical role of electrode design and material engineering in developing cost-effective, scalable solutions for monitoring pharmaceutical contaminants in environmental and biological systems.

## Supplementary Material



## Notes

Cyclic voltammetry cleaning test of CPME-BCA; SEM photomicrographs, EDS spectra, and FTIR spectrum of BC; contact angle measurements for unmodified and modified CPEs; the proposed oxidation mechanism of SFD; the proposed interactions between the BCA surface and SFD molecules; experimental optimization data for the voltammetric response of CPME-BCA and SPCE-BCA; SFD species distribution graph; evaluation of concomitant species for SFD determination; experimental parameters tested for sulfanilamide detection; ANOVA and Tukey test results are provided for contact angle and peak currents obtained from CPE and SPCE electrodes; and RSD values of relative peak currents for 5.0 μmol L^–1^ of SFD in the presence of concomitant species at three concentration levels (PDF)

## References

[1] Lisboa T. P., Alves G. F., de Faria L. V., de Souza C. C., Matos M. A. C., Matos R. C. (2022). 3D-Printed Electrode an Affordable Sensor for Sulfanilamide
Monitoring in Breast Milk, Synthetic Urine, and Pharmaceutical Formulation
Samples. Talanta.

[2] Di-Oliveira M., Araújo D. A. G., Ramos D. L. O., De Faria L. V., Rocha R. G., Sousa R. M. F., Munoz R. A. A. (2024). Sequential Cyclic-Square-Wave
Voltammetric Determination of Sulfanilamide and Ciprofloxacin in Environment
Water Samples Using a 3D-Printed Electrochemical Device. Electrochim. Acta.

[3] de
Faria L. V., Lisboa T. P., Campos N. D. S., Alves G. F., Matos M. A. C., Matos R. C., Munoz R. A. A. (2021). Electrochemical
Methods for the Determination of Antibiotic Residues in Milk: A Critical
Review. Anal. Chim. Acta.

[4] Zhang Y., Lv Y., Chen Y., Li Y., Wang Y., Zhao H. (2022). Trimetallic
Ag@Pt-Rh Core-Shell Nanocubes Modified Anode for Voltammetric Sensing
of Dopamine and Sulfanilamide. Chem. Eng. Sci..

[5] de
Moura Junior F. G., de M., Veloso W. B., de Oliveira
Junior J. A., Kraatz H. B., da Silva I. S., Dantas L. M. F. (2021). Voltammetric
Determination of Sulfanilamide Using a Cobalt Phthalocyanine Chitosan
Composite. Monatsh. Chem..

[6] Rajendiran N., Thulasidhasan J. (2015). Interaction
of Sulfanilamide and Sulfamethoxazole with
Bovine Serum Albumin and Adenine: Spectroscopic and Molecular Docking
Investigations. Spectrochim. Acta, Part A.

[7] Kim Y. R., Park S., Kim J. Y., Choi J. D., Moon G. I. (2024). Simultaneous
Determination of 31 Sulfonamide Residues in Various Livestock Matrices
Using Liquid Chromatography-Tandem Mass Spectrometry. Appl. Biol. Chem..

[8] Li S., Zhang C., Tang H. X., Gu Y., Guo A. J., Wang K., Lian K. Q. (2023). Determination of 24 Sulfonamide Antibiotics
in Instant Pastries by Modified QuEChERS Coupled with Ultra Performance
Liquid Chromatography-Tandem Mass Spectrometry. J. Food Drug Anal..

[9] Jamal S., Baderia V. K., Agrawal Y. K., Sanghi S. K. (2019). Fluorescence Detection
and Identification of Eight Sulphonamides Using Capillary Electrophoresis
on Released Excipients in Lake Water. Arab.
J. Chem..

[10] Font G., Juan-García A., Picó Y. (2007). Pressurized
Liquid Extraction Combined
with Capillary Electrophoresis-Mass Spectrometry as an Improved Methodology
for the Determination of Sulfonamide Residues in Meat. J. Chromatogr. A.

[11] Darweesh S. A. (2016). Simultaneous
Determination of Sulfanilamide and Furosemide by Using Derivative
Spectrophotometry. Ibn Al-Haitham J. Pure Appl.
Sci..

[12] Errayess S. A., Lahcen A. A., Idrissi L., Marcoaldi C., Chiavarini S., Amine A. (2017). A Sensitive Method for the Determination
of Sulfonamides in Seawater Samples by Solid Phase Extraction and
UV–Visible Spectrophotometry. Spectrochim.
Acta - Part A.

[13] Tadi K. K., Motghare R. V., Ganesh V. (2014). Electrochemical Detection
of Sulfanilamide
Using Pencil Graphite Electrode Based on Molecular Imprinting Technology. Electroanalysis.

[14] de
Faria L. V., Lisboa T. P., Matias T. A., de Sousa R. A., Matos M. A. C., Munoz R. A. A., Matos R. C. (2021). Use of Reduced Graphene
Oxide for Sensitive Determination of Sulfanilamide in Synthetic Biological
Fluids and Environmental Samples by Batch Injection Analysis. J. Electroanal. Chem..

[15] Cancelliere R., Cianciaruso M., Carbone K., Micheli L. (2022). Biochar: A Sustainable
Alternative in the Development of Electrochemical Printed Platforms. Chemosensors.

[16] de
Almeida L. S., Oreste E. Q., Maciel J. V., Heinemann M. G., Dias D. (2020). Electrochemical Devices Obtained from Biochar: Advances in Renewable
and Environmentally-Friendly Technologies Applied to Analytical Chemistry. Trends Environ. Anal. Chem..

[17] Jadon N., Jain R., Sharma S., Singh K. (2016). Recent Trends in Electrochemical
Sensors for Multianalyte Detection – A Review. Talanta.

[18] Kuntoji G., Kousar N., Gaddimath S., Sannegowda L. K.. (2024). Macromolecule–Nanoparticle-Based
Hybrid Materials for Biosensor Applications. Biosensors.

[19] Kalinke C., de Oliveira P. R., Mangrich A. S., Marcolino-Junior L. H., Bergamini M. F. (2020). Chemically-Activated Biochar from Ricinus Communis
L. Cake and Their Potential Applications for the Voltammetric Assessment
of Some Relevant Environmental Pollutants. J.
Braz. Chem. Soc..

[20] Lehmann, J. ; Joseph, S. Biochar for Environmental Management: Science and Technology; Earthscan: London & Sterling, VA: London, 2009.

[21] Saravanan A., Kumar P. S. (2022). Biochar
Derived Carbonaceous Material for Various Environmental
Applications: Systematic Review. Environ. Res..

[22] Cha J. S., Park S. H., Jung S. C., Ryu C., Jeon J. K., Shin M. C., Park Y. K. (2016). Production and Utilization
of Biochar:
A Review. J. Ind. Eng. Chem..

[23] Cabral L. L., Bottini R. C. R., Gonçalves A. J., Junior M. M., Rizzo-Domingues R. C.
P., Lenzi M. K., Nagalli A., Passig F. H., dos Santos P. M., de Carvalho K. Q. (2025). Food Dye Adsorption in Single and Ternary Systems by
the Novel Passion Fruit Peel Biochar Adsorbent. Food Chem..

[24] Kamarudin N. S., Dahalan F. A., Hasan M., An O. S., Parmin N. A., Ibrahim N., Hamdzah M., Zain N. A. M., Muda K., Wikurendra E. A. (2022). Biochar: A Review of Its History, Characteristics,
Factors That Influence Its Yield, Methods of Production, Application
in Wastewater Treatment and Recent Development. Biointerface Res. Appl. Chem..

[25] Zhang L., Ren Y., Xue Y., Cui Z., Wei Q., Han C., He J. (2020). Preparation of Biochar by Mango Peel
and Its Adsorption Characteristics
of Cd­(Ii) in Solution. RSC Adv..

[26] Meng J., Tao M., Wang L., Liu X., Xu J. (2018). Changes in Heavy Metal
Bioavailability and Speciation from a Pb-Zn Mining Soil Amended with
Biochars from Co-Pyrolysis of Rice Straw and Swine Manure. Sci. Total Environ..

[27] Yang Z., Yang R., Dong G., Xiang M., Hui J., Ou J., Qin H. (2021). Biochar Nanocomposite
Derived from Watermelon Peels
for Electrocatalytic Hydrogen Production. ACS
Omega.

[28] Gan S., Chen B., Li L., Sushkova S., Garg A. (2024). Effect of
Three Different Types of Biochar on Bioelectricity Generated from
Plant Microbial Fuel Cells under Unsaturated Soil Condition. ACS Appl. Bio Mater..

[29] Hsu C. H., Pan Z. B., Chen C. R., Wei M. X., Chen C. A., Lin H. P., Hsu C. H. (2020). Synthesis of Multiporous Carbons
from the Water Caltrop Shell for High-Performance Supercapacitors. ACS Omega.

[30] Kalinke C., De Oliveira P. R., Bonacin J. A., Janegitz B. C., Mangrich A. S., Marcolino-Junior L. H., Bergamini M. F. (2021). State-of-the-Art and Perspectives
in the Use of Biochar for Electrochemical and Electroanalytical Applications. Green Chem..

[31] Li Y., Xu R., Wang H., Xu W., Tian L., Huang J., Liang C., Zhang Y. (2022). Recent Advances
of Biochar-Based
Electrochemical Sensors and Biosensors. Biosensors.

[32] Zheng Y., Yu C., Fu L. (2023). Biochar-Based Materials for Electroanalytical Applications:
An Overview. Green Anal. Chem..

[33] Valenga M. G. P., Gevaerd A., Marcolino-Junior L. H., Bergamini M. F. (2024). Biochar
from Sugarcane Bagasse: Synthesis, Characterization, and Application
in an Electrochemical Sensor for Copper (II) Determination. Biomass and Bioenergy.

[34] Oliveira P. R., Lamy-Mendes A. C., Rezende E. I. P., Mangrich A. S., Marcolino
Junior L. H., Bergamini M. F. (2015). Electrochemical Determination of
Copper Ions in Spirit Drinks Using Carbon Paste Electrode Modified
with Biochar. Food Chem..

[35] Kalinke C., Mangrich A. S., Marcolino-Junior L. H., Bergamini M. F. (2016). Biochar
Prepared from Castor Oil Cake at Different Temperatures: A Voltammetric
Study Applied for Pb2+, Cd2+ and Cu2+ Ions Preconcentration. J. Hazard. Mater..

[36] Oliveira G. A., Gevaerd A., Mangrich A. S., Marcolino-Junior L. H., Bergamini M. F. (2021). Biochar Obtained from Spent Coffee Grounds: Evaluation
of Adsorption Properties and Its Application in a Voltammetric Sensor
for Lead (II) Ions. Microchem. J..

[37] Kalinke C., Mangrich A. S., Marcolino-Junior L. H., Bergamini M. F. (2016). Carbon
Paste Electrode Modified with Biochar for Sensitive Electrochemical
Determination of Paraquat. Electroanalysis.

[38] de
Oliveira P. R., Kalinke C., Gogola J. L., Mangrich A. S., Junior L. H. M., Bergamini M. F. (2017). The Use of Activated Biochar for
Development of a Sensitive Electrochemical Sensor for Determination
of Methyl Parathion. J. Electroanal. Chem..

[39] Sant’Anna M. V.
S., Carvalho S. W. M. M., Gevaerd A., Silva J. O. S., Santos E., Carregosa I. S. C., Wisniewski A., Marcolino-Junior L. H., Bergamini M. F., Sussuchi E. M. (2020). Electrochemical
Sensor Based on Biochar and Reduced Graphene Oxide Nanocomposite for
Carbendazim Determination. Talanta.

[40] Kalinke C., de Oliveira P. R., San Emeterio M. B., González-Calabuig A., del Valle M., Mangrich A. S., Marcolino-Junior L. H., Bergamini M. F. (2019). Voltammetric
Electronic Tongue Based on Carbon Paste
Electrodes Modified with Biochar for Phenolic Compounds Stripping
Detection. Electroanalysis.

[41] Kalinke C., Zanicoski-Moscardi A. P., de Oliveira P. R., Mangrich A. S., Marcolino-Junior L. H., Bergamini M. F. (2020). Simple
and Low-Cost Sensor Based on Activated Biochar for the Stripping Voltammetric
Detection of Caffeic Acid. Microchem. J..

[42] Valenga M. G. P., Martins G., Martins T. A. C., Didek L. K., Gevaerd A., Marcolino-Junior L. H., Bergamini M. F. (2023). Biochar: An Environmentally Friendly
Platform for Construction of a SARS-CoV-2 Electrochemical Immunosensor. Sci. Total Environ..

[43] Dong X., He L., Liu Y., Piao Y. (2018). Preparation of Highly Conductive
Biochar Nanoparticles for Rapid and Sensitive Detection of 17β-Estradiol
in Water. Electrochim. Acta.

[44] Morawski F., de M., Ferreira L. M. C., Jost C. L., Bergamini M. F., Marcolino-Junior L. H. (2024). Silica
Graphite as an Ink Additive for Stencil Printed
Electrodes: A Novel Approach for Electroanalytical Determination of
Sulfanilamide. J. Electroanal. Chem..

[45] Rocha R. G., De Faria L. V., Silva V. F., Muñoz R. A. A., Richter E. M. (2023). Carbon Black Integrated Polylactic
Acid Electrodes
Obtained by Fused Deposition Modeling: A Powerful Tool for Sensing
of Sulfanilamide Residues in Honey Samples. J. Agric. Food Chem..

[46] Ferraz B. R. L., Guimarães T., Profeti D., Profeti L. P. R. (2018). Electrooxidation
of Sulfanilamide and Its Voltammetric Determination in Pharmaceutical
Formulation, Human Urine and Serum on Glassy Carbon Electrode. J. Pharm. Anal..

[47] Miller, J. N. ; Miller, J. C. Statistics and Chemometrics for Analytical Chemistry, 6th ed.; Pearson Education Limited: Gosport, 2010.

[48] Baccarin M., Cervini P., Cavalheiro E. T. G. (2018). Comparative
Performances of a Bare
Graphite-Polyurethane Composite Electrode Unmodified and Modified
with Graphene and Carbon Nanotubes in the Electrochemical Determination
of Escitalopram. Talanta.

[49] Dong J., Wu Y., Wang C., Lu H., Li Y. (2020). Three-Dimensional Electrodes
Enhance Electricity Generation and Nitrogen Removal of Microbial Fuel
Cells. Bioprocess Biosyst. Eng..

[50] Wu C., Huang L., Xue S. G., Huang Y. Y., Hartley W., Cui M. Q., Wong M. H. (2017). Arsenic Sorption by Red Mud-Modified
Biochar Produced from Rice Straw. Environ. Sci.
Pollut. Res..

[51] Vijayaraghavan K. (2021). The Importance
of Mineral Ingredients in Biochar Production, Properties and Applications. Crit. Rev. Environ. Sci. Technol..

[52] Akbari A., Peighambardoust S. J., Kazemian H. (2025). Comparative Study on the Impact of
Physicochemical Characteristics of the Activated Carbons Derived from
Biochar/Hydrochar on the Adsorption Performances. Environ. Res..

[53] Suman S., Yadav A., Jain T., SK A. A. (2021). Study in
the Changes
on the Functional Groups Present in Biomass during Pyrolysis Process. IOP Conf. Ser.:Mater. Sci. Eng..

[54] Ternero-Hidalgo J. J., Rosas J. M., Palomo J., Valero-Romero M. J., Rodríguez-Mirasol J., Cordero T. (2016). Functionalization
of
Activated Carbons by HNO_3_ Treatment: Influence of Phosphorus
Surface Groups. Carbon.

[55] Vinke P., van der Eijk M., Verbree M., Voskamp A. F., van Bekkum H. (1994). Modification
of the Surfaces of a Gasactivated Carbon and a Chemically Activated
Carbon with Nitric Acid, Hypochlorite, and Ammonia. Carbon.

[56] Jayakumar A., Krishnan A. (2025). Exploring Surface Hydrophilicity
and Hydrophobicity:
Influence of Copper Deposition on TiO2 Nanotubes. Mater. Lett..

[57] Xu T., Du J., Zhang J., David W., Liu P., Faheem M., Zhu X., Yang J., Bao J. (2022). Microbially-Mediated
Synthesis of
Activated Carbon Derived from Cottonseed Husks for Enhanced Sulfanilamide
Removal. J. Hazard. Mater..

[58] Calegari F., de Souza L. P., Barsan M. M., Brett C. M. A., Marcolino-Junior L. H., Bergamini M. F. (2017). Construction
and Evaluation of Carbon Black and Poly­(Ethylene
Co-Vinyl)­Acetate (EVA) Composite Electrodes for Development of Electrochemical
(Bio)­Sensors. Sens. Actuators, B.

[59] Zhang K., Ge Y., He S., Ge F., Huang Q., Huang Z., Wang X., Wen Y., Wang B. (2020). Development of New
Electrochemical Sensor Based on Kudzu Vine Biochar Modified Flexible
Carbon Electrode for Portable Wireless Intelligent Analysis of Clenbuterol. Int. J. Electrochem. Sci..

[60] Allende S., Liu Y., Jacob M. V. (2024). Electrochemical
Sensing of Paracetamol Based on Sugarcane
Bagasse-Activated Biochar. Ind. Crops Prod..

[61] Zappi D., Varani G., Cozzoni E., Iatsunskyi I., Laschi S., Giardi M. T. (2021). Innovative Eco-Friendly
Conductive
Ink Based on Carbonized Lignin for the Production of Flexible and
Stretchable Bio-Sensors. Nanomaterials.

[62] Ganesan S., Sivam S., Elancheziyan M., Senthilkumar S., Ramakrishan S. G., Soundappan T., Ponnusamy V. K. (2022). Novel Delipidated
Chicken Feather Waste-Derived Carbon-Based Molybdenum Oxide Nanocomposite
as Efficient Electrocatalyst for Rapid Detection of Hydroquinone and
Catechol in Environmental Waters. Environ. Pollut..

[63] Nikhil N., Srivastava S. K., Srivastava A., Srivastava M., Prakash R. (2022). Electrochemical Sensing
of Roxarsone on Natural Biomass-Derived
Two-Dimensional Carbon Material as Promising Electrode Material. ACS Omega.

[64] Kalinke C., Wosgrau V., Oliveira P. R., Oliveira G. A., Martins G., Mangrich A. S., Bergamini M. F., Marcolino-Junior L. H. (2019). Green Method
for Glucose Determination Using Microfluidic Device with a Non-Enzymatic
Sensor Based on Nickel Oxyhydroxide Supported at Activated Biochar. Talanta.

[65] Chen S., Wang C., Zhang M., Zhang W., Qi J., Sun X., Wang L., Li J. (2020). N-Doped Cu-MOFs for
Efficient Electrochemical
Determination of Dopamine and Sulfanilamide. J. Hazard. Mater..

[66] Li H., Kuang X., Shen X., Zhu J., Zhang B., Li H. (2019). Improvement of Voltammetric Detection of Sulfanilamide with a Nanodiamond-Modified
Glassy Carbon Electrode. Int. J. Electrochem.
Sci..

[67] Ferraz B. R. L., Profeti D., Profeti L. P. R. (2018). Sensitive Detection of Sulfanilamide
by Redox Process Electroanalysis of Oxidation Products Formed in Situ
on Glassy Carbon Electrode. J. Solid State Electrochem..

[68] He B. S., Yan X. H. (2018). Modifications
of Au Nanoparticle-Functionalized Graphene
for Sensitive Detection of Sulfanilamide. Sensors.

