# Supplementary material for: Biochar for Electrochemical Sulfanilamide Detection: Comparative Evaluation of Carbon Paste and Screen-Printed Electrodes
Source: ACS Omega. 2025 Jul 28;10(30):33595–606. doi: 10.1021/acsomega.5c04276 (PMC12332590; doi:10.1021/acsomega.5c04276)
Supplement: Supplementary file 1 [file ao5c04276_si_001.pdf]

Supplementary Material:

Biochar for Electrochemical Sulfanilamide Detection: Comparative Evaluation of Carbon Paste and Screen-Printed Electrodes

Lucas L. Cabral[a], Cristiane Kalinke[a,b*], Marcia G. P. Valenga[a], Luiz H. Marcolino-Junior[a], and Márcio F. Bergamini[a]

[a] *Laboratory of Electrochemical Sensors (LabSensE), Department of Chemistry, Federal University of Paraná, 81531-980, Curitiba, PR, Brazil.*

[b] *Institute of Advanced Materials (INAM), University Jaume I, 12006, Castellon de la Plana, Spain.*

Summary

Fig. S1. Cyclic voltammetry cleaning test of CPME-BCA electrode in the determination of SFD 50 µmol L^{-1}. CPME-BCA 25 wt%; preconcentration: B-R buffer, pH 2.0; supporting electrolyte: B-R buffer, pH 6.0; and preconcentration time: 5 min. 3

Fig. S2. (A) SEM photomicrographs for BC at magnification of 2000 x, (B) EDS spectra for BC and BCA, (C) FTIR spectrum for BC, and (D) contact angle for carbon paste electrodes. .5

Fig. S3. Sulfanilamide oxidation mechanism. 6

Fig. S4. Proposed interaction mechanisms between the BCA surface and SFD molecules.......7

Experimental optimization for voltammetric response of CPME-BCA and SPCE-BCA 8

Fig. S5. Experimental optimized parameters for SFD determination by CPME-BCA: (A) biochar proportion in the carbon paste, (B) preconcentration solution pH, (C) measurement

solution pH, and (D) preconcentration time; and by SPCE-BCA: (E) biochar proportion in the dispersion for drop-casting, and (F) preconcentration time. 11

Fig. S6. Sulfanilamide distribution species graph. 12

Fig. S7. Evaluation of concomitant species for SFD determination using the SPCE-BCA. Preconcentration solution containing the concomitant species: B-R buffer, pH 2.0; Supporting electrolyte: B-R buffer, pH 6.0. 13

Table S1. Experimental parameters evaluated for the determination of sulfanilamide. 14

Table S2. Variance analysis (ANOVA) and Tukey test of the contact angle obtained from the CPEs and SPCEs electrodes. 15

Table S3. Variance analysis (ANOVA) and Tukey test of the anodic peak currents (I_{pa}) obtained from the CPEs and SPCEs electrodes. 16

Table S4. RSD of relative peak currents for 5.0 μmol L^{-1} of SFD in the presence of concomitant species at three different concentration levels (0.5, 5.0, and 50 μmol L^{-1}). 17

References 18

* Corresponding authors: luiz1berto@ufp.br (LHM); bergamini@ufpr.br (MB).

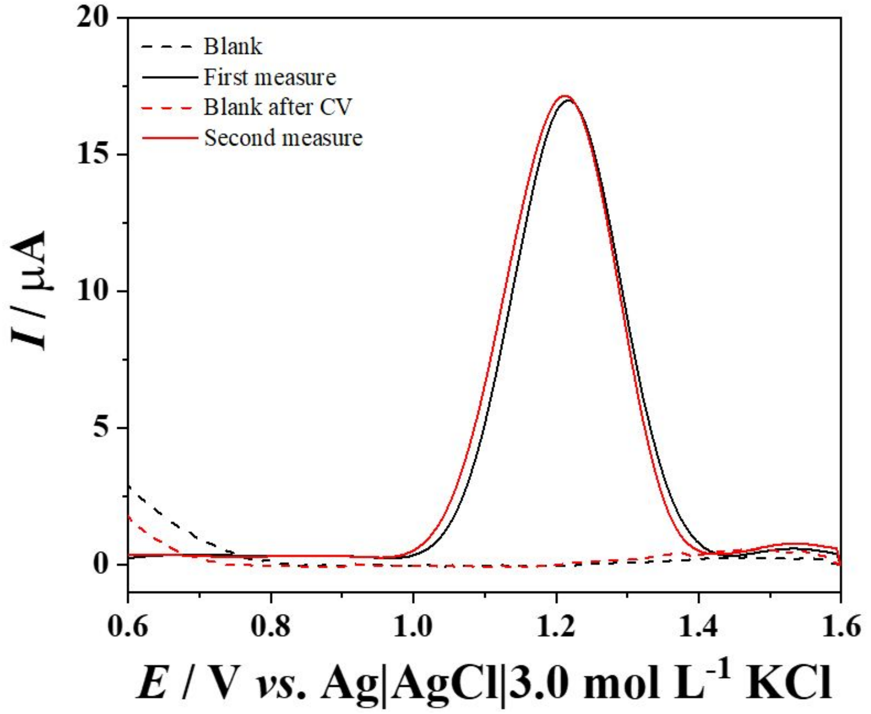

Fig. S1. Cyclic voltammograms before (first measurement) and after cleaning (second measurement) test using the CPME-BCA electrode for the determination of 50 μmol L^{-1} SFD. Biochar proportion: 25 wt%, preconcentration: B-R buffer pH 2.0, supporting electrolyte: B-R buffer pH 6.0, and preconcentration time: 5 min.

Based on voltammogram results, it was observed that no faradaic signal was detected in the initial blank measurement. After preconcentration of the SFD and the first measurement, an oxidation signal appeared close to +1.2 V, indicating the presence of the analyte. After the measurement and the subsequent electrochemical cleaning step performed by cyclic voltammetry (CV), the complete removal of the SFD from the electrode surface was confirmed, as no faradaic signal was detected in the second blank measurement. In the subsequent measurement (measurement 2), an oxidation signal with characteristics similar to the first measurement was again observed, demonstrating the reproducibility of the detection. Therefore, electrochemical cleaning via CV in 0.1 mol L^{-1} B-R buffer solution at pH 6.0 was

adopted for all following experiments. A maximum of three measurements per electrode were performed before the electrode surface was renewed by polishing, since the signal diminishes.

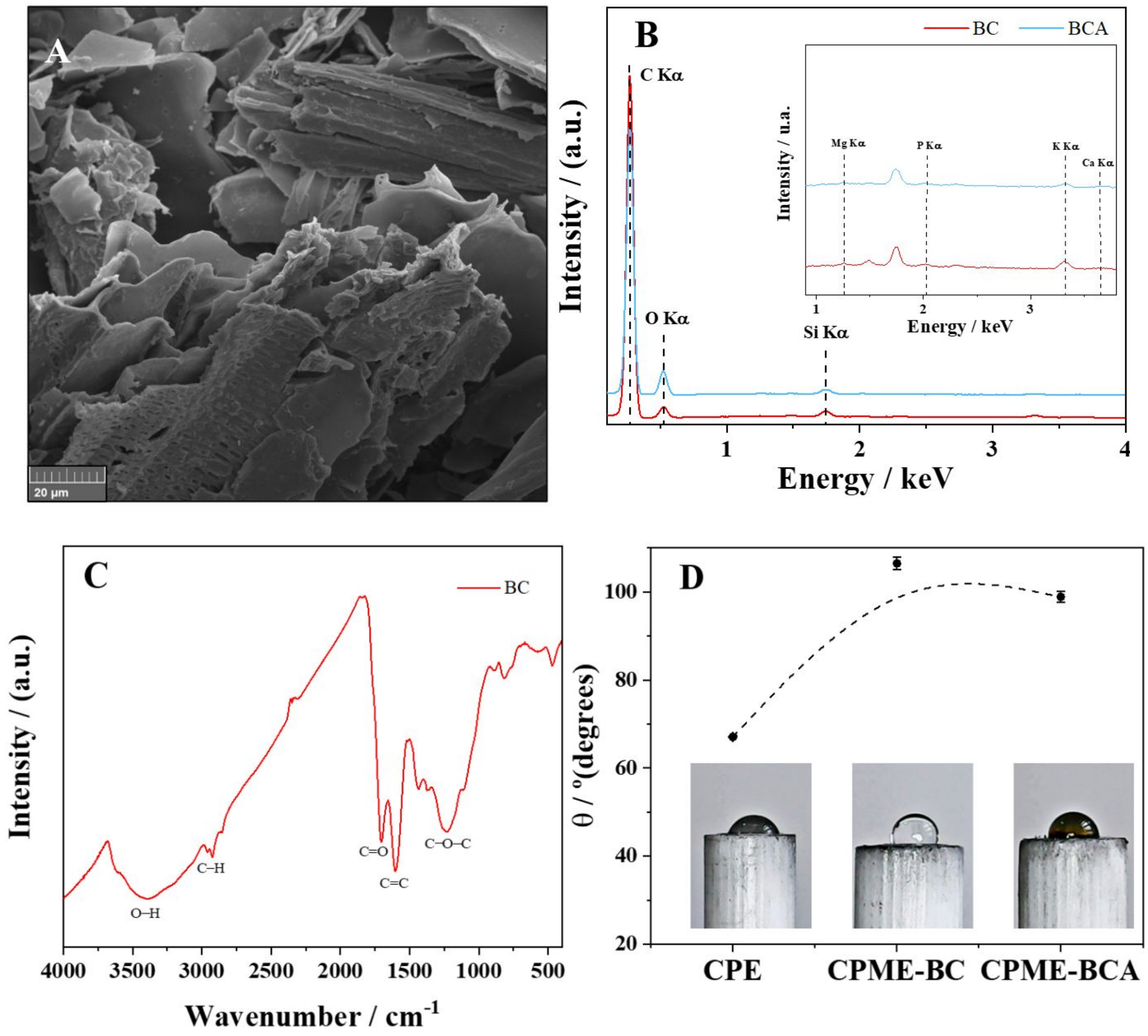

Fig. S2. (A) SEM photomicrographs for BC at magnification of 2000 x, (B) EDS spectra for BC and BCA, (C) FTIR spectrum for BC, and (D) contact angle for carbon paste electrodes.

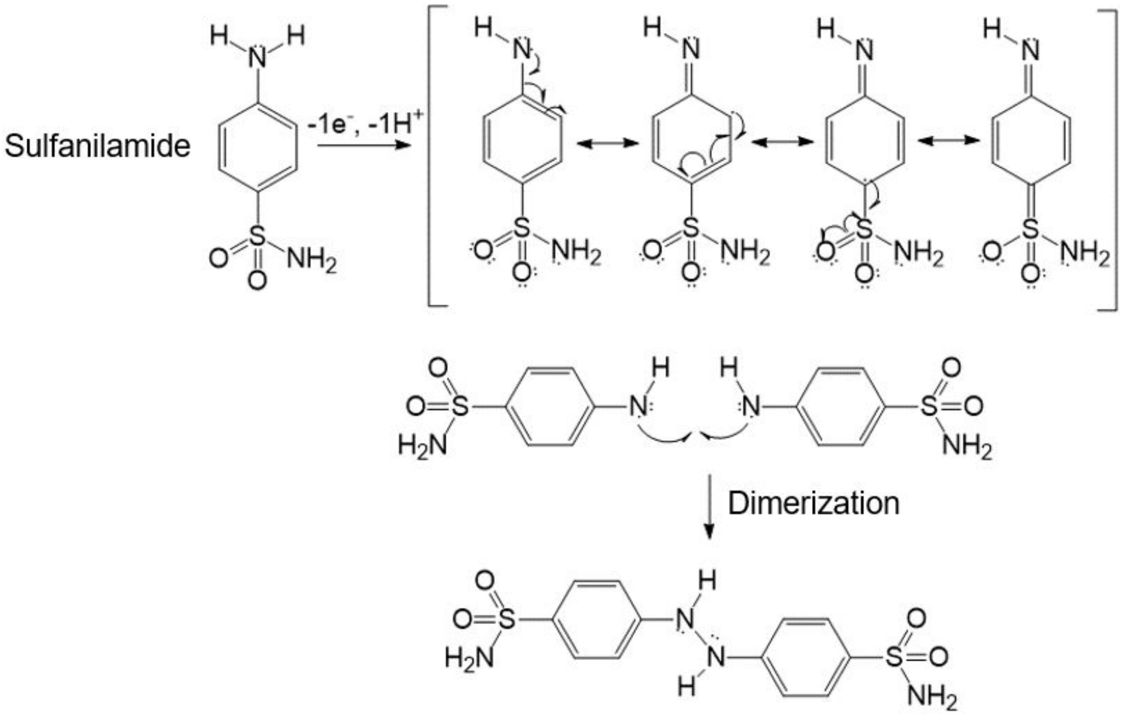

Fig. S3. Sulfanilamide oxidation mechanism.

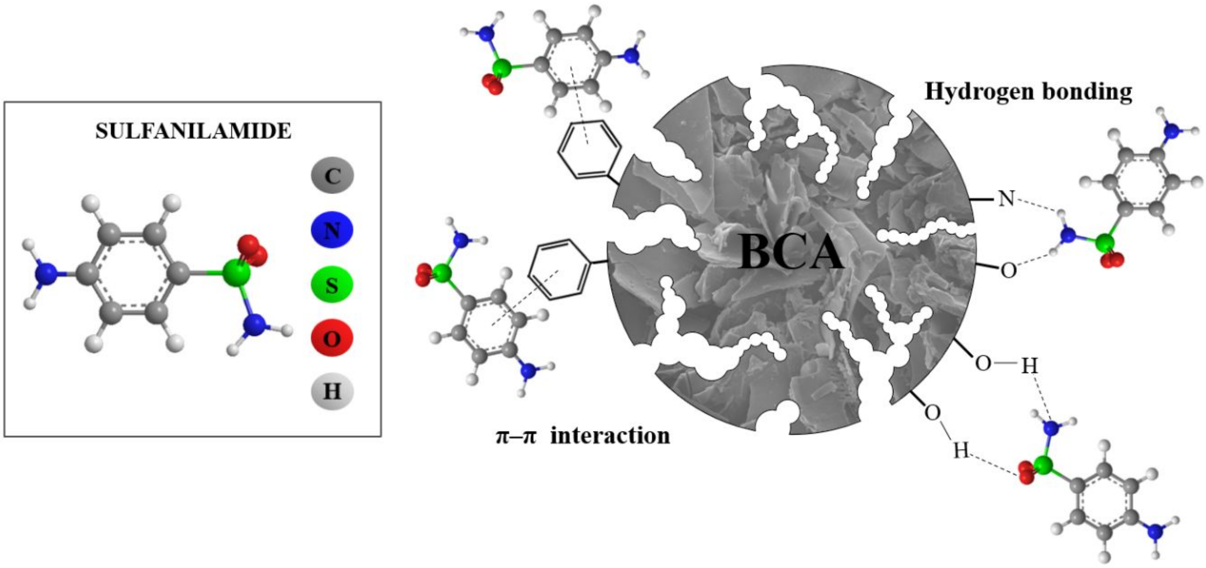

Fig. S4. Proposed interaction mechanisms between the BCA surface and SFD molecules.

Experimental optimization for voltammetric response of CPME-BCA and SPCE-BCA

The experimental parameters for SFD determination using CPME-BCA and SPCE-BCA electrodes were evaluated to provide better voltammetric responses. Table S1 presents the results of the experimental optimization. For the CPME-BCA, firstly, the biochar proportion in the carbon paste was evaluated since it can directly affect the voltammetric response of the sensor[1] by providing more adsorptive sites for analyte interaction in the electrode surface, enhancing its preconcentration. From Fig. S5A, a maximum peak current is noted for 25 wt% of BCA in the carbon paste and a decrease for values above 30 wt%. This can be explained by the loss of conductivity in the electrode surface due to the high proportion of biochar, an amorphous and insulating material, at the expense of a reduced graphite proportion, which is a crystalline and conductive material. Therefore, the optimum proportions of mineral oil, graphite, and BCA in the carbon paste were 25, 50, and 25 wt%, respectively, since it combines good SFD preconcentration and improved response signal.

The pH of the SFD preconcentration solution (B-R buffer) resulted in a higher anodic peak current under acidic conditions for the analyte oxidation, with best results at pH 2.0 (Fig. S5B). The species distribution for SFD helps to understand the analyte behavior (Fig. S6). Under acidic conditions, the sulfanilamide present in the solution is protonated or neutral, and specifically at pH lower than 2.0, the predominance of protonated species is indicative of the occurrence of electrostatic attraction with the positive charge in the SFD amino groups with the negative charge of groups present on the biochar surface. Preconcentration was performed in a different solution from the analysis solution (ex-situ) aiming to promote the accumulation of SFD on biochar, exploring its interaction abilities and improving the signal response. Also, the ex-situ analysis has some advantages, such as minimizing interference, increasing sensitivity and selectivity, and better control of the experimental conditions of the analysis[2].

The pH of the analysis solution (B-R buffer) is another critical experimental parameter since the voltammetric measurements were obtained separately from the preconcentration solution in another electrochemical cell. Better results were observed at neutral pH, with 6.0 considered the best condition (Fig. S5C). The lower concentration of H^+ ions present in the solution can favor the deprotonation of biochar carboxylic groups, displacing sulfanilamide from the electrode surface; a similar behavior was described by Kalinke et al.[3] in caffeic acid determination using carbon paste electrode modified with castor oil cake biochar.

The preconcentration time was evaluated from 1.0 to 15 min, and the accumulation step was performed in a solution containing 50 μmol L^{-1} of sulfanilamide. As shown in Fig. S5D, the voltammetric signals increased with the increase of pre-concentration time until 10 min. After 10 min, the peak current decreased, indicating the saturation of available biochar sites harming the interaction between BCA and SFD. Although 10 min registered the higher I_{pa}, the difference for 5 min is slight (2.68 μA) with the standard deviation approximating the mean peak current for both times. Thus, 5 min was adopted as the best pre-concentration time since the analytical frequency is not affected for complete measurements in less than 10 min[3].

Regarding SPCE-BCA, the same pH values of B-R buffer solutions were studied and optimized, being pH 2.0 and 7.0 for the preconcentration and analysis solutions, respectively. The biochar proportion in the dispersion and the preconcentration time were also evaluated. From the results of biochar proportion in the dispersion (Fig. S5E), it was possible to observe an increase in the peak current value until the dispersion of BCA of 1.0 mg mL^{-1}, probably due to the higher amount of biochar on the electrode surface, providing a better interaction with SFD. At the proportion of 1.5 mg mL^{-1}, the decrease in the peak current and the reduced analytical signal may be attributed to a difficulty in the electron transfer between the electrode and solution. This effect is possibly caused by the increased amount of BCA on the working electrode surface, since biochar is a non-conductive material, and similar behavior was

observed for CPME-BCA. Other authors also reported the use of a proportion of 1.0 mg mL^{-1} of biochar from different biomasses in the dispersion for modification of GCEs and SPEs by drop-casting[4–6]. The proportion of 1.0 mg mL^{-1} of BCA was maintained for further steps.

The accumulation step in the SPCE-BCA was investigated in a solution containing 5.0 μmol L^{-1} of SFD with preconcentration time varying from 1.0 to 10 min. In Fig. S5F, it is possible to verify an increase in preconcentration time until 2 min (I_{pa} = 1.76±0.26 μA) have passed. For times exceeding 2 min, a decrease in the voltammetric signal was observed. This behavior is due to the progressive saturation of active sites within the biochar, limiting the availability of adsorptive sites for the analyte, thereby reducing the interaction between the BCA and SFD molecules. Therefore, 2 min was assumed as the best time for preconcentrate SFD in the SPCE-BCA surface, which is considered a short time for analytical determination procedures.

Comparing the distinct preconcentration times of SFD for CPME-BCA and SPCE-BCA, some factors help explain these behaviors. Firstly, different methodologies were employed for each electrode during the preconcentration time tests. For CPME-BCA, dynamic preconcentration was performed by constantly agitating the SFD solution in contact with the electrode surface. Conversely, for SPCE-BCA, static preconcentration (without agitation) was used, with the SFD solution dropped on the working electrode surface. Moreover, different analyte concentrations were used in the tests for CPME-BCA (50 μmol L^{-1} SFD) and SPCE-BCA (5.0 μmol L^{-1} SFD), which may have also contributed to variations in preconcentration. Another key factor is the likely greater amount of biochar present on the surface of CPME-BCA compared to SPCE-BCA, which could facilitate higher analyte accumulation over time. Combining these factors helps to understand the differences observed in the preconcentration time performance of the electrodes.

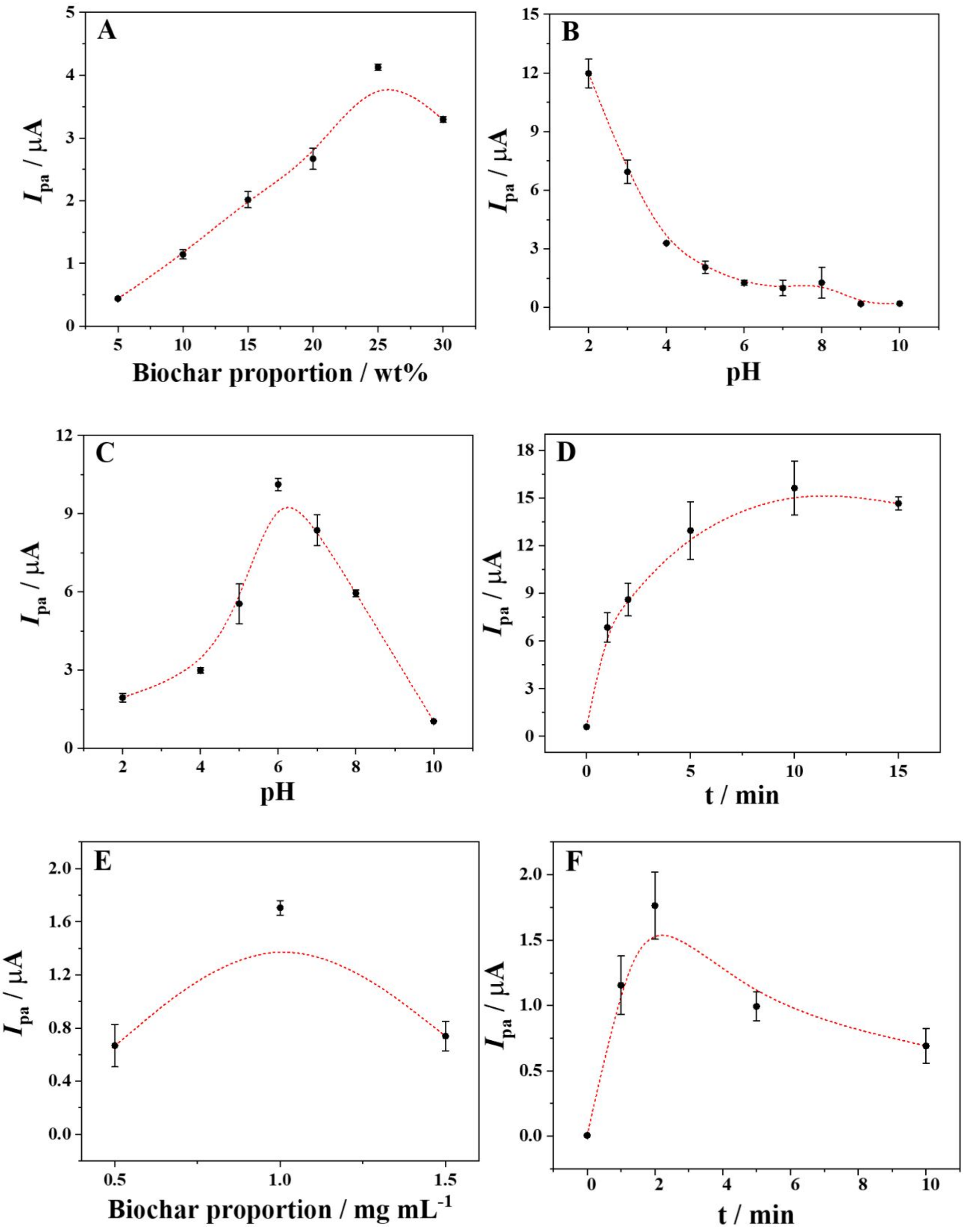

Fig. S5. Experimental optimized parameters for SFD determination by CPME-BCA: (A) biochar proportion in the carbon paste, (B) preconcentration solution pH, (C) measurement solution pH, and (D) preconcentration time; and by SPCE-BCA: (E) biochar proportion in the dispersion for drop-casting, and (F) preconcentration time.

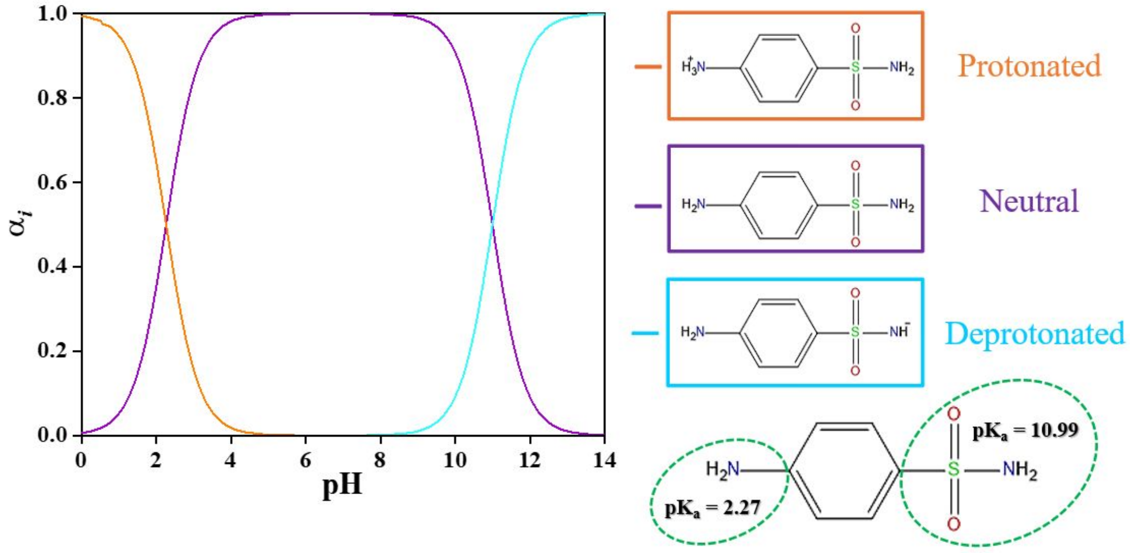

Fig. S6. Sulfanilamide distribution species graph.

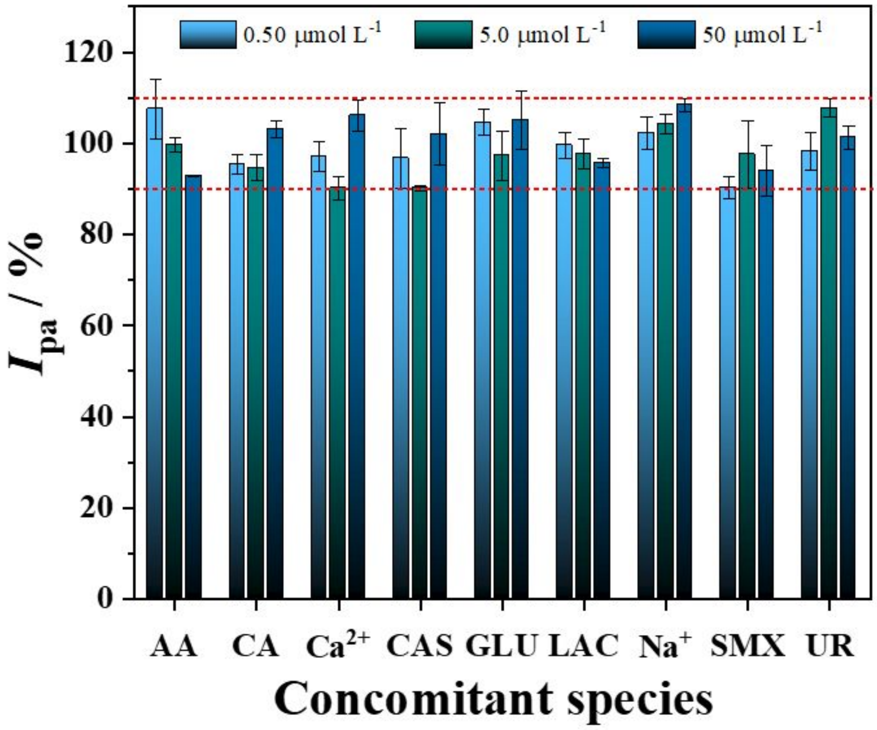

Fig. S7. Evaluation of concomitant species for SFD determination using the SPCE-BCA. Preconcentration solution containing concomitant species: B-R buffer pH 2.0, supporting electrolyte: B-R buffer pH 6.0.

Table S1. Experimental parameters evaluated for the determination of sulfanilamide.

Sensor	Parameter	Evaluated condition	Optimized
CPME-BCA	Biochar proportion	0 – 30 wt%	25 wt%
	Measurement solution	pH 2.0 – 10	pH 2.0
	Preconcentration solution	pH 2.0 – 10	pH 6.0
	Preconcentration time	0 – 15 min	5 min
SPCE-BCA	Biochar proportion	0.5 – 1.5 mg mL^{-1}	1.0 mg mL^{-1}
	Preconcentration time	0 – 10 min	2 min

Table S2. Variance analysis (ANOVA) and Tukey test of the contact angle obtained from the CPEs and SPCEs electrodes.

ANOVA						
**Variable response**	**Effect**	**SQ**	**DF**	**MQ**	F_{calc}	F_{crit}
	Model	1754	2	877.3	739.6	9.55
θ for CPEs	Residuals	3.56	3	1.19		
	Total	1758	5			
	Model	175.1	2	87.57	15.82	9.55
θ for SPCEs	Residuals	16.61	3	5.54		
	Total	191.7	5			

Tukey Test				
**Electrode**	**θ (º)**	**1**	**2**	**3**
CPE	67.1 ± 0.2	A		
CPME-BC	106 ± 1		B	
CPME-BCA	98.9 ± 1.3			**C**
SPCE	103 ± 1	A		
SPCE-BC	104 ± 4	A		
SPCE-BCA	92.0 ± 1.7		B	

θ = water contact angle (º); SQ = sum of squares; DF = degree of freedom; MQ = mean square; F_{calc} = Fisher distribution (calculated); F_{crit} = Fisher distribution (tabulated with a 5% of probability); A, B, and C = electrodes that do not share a letter significantly differ.

Table S3. Variance analysis (ANOVA) and Tukey test of the anodic peak currents (I_{pa}) obtained from the CPEs and SPCEs electrodes.

ANOVA

**Variable response**	**Effect**	**SQ**	**DF**	**MQ**	**F_{calc}**	**F_{crit}**
I_{pa} for CPEs	Model	10.7	2	5.35	151	5.14
	Residuals	0.21	6	0.03		
	Total	10.9	8			
I_{pa} for SPCEs	Model	1.00	2	0.50	19.0	5.14
	Residuals	0.16	6	0.03		
	Total	1.16	8			

Tukey Test

**Electrode**	**I_{pa} (μA)**	**1**	**2**
CPE	0.11 ± 0.01	A	
CPME-BC	0.12 ± 0.01	A	
CPME-BCA	2.5 ± 0.3		B
SPCE	1.2 ± 0.1	A	
SPCE-BC	0.91 ± 0.22	A	
SPCE-BCA	1.7 ± 0.07		B

I_{pa} for CPEs = peak current for carbon paste electrodes (μA); I_{pa} for SPCEs = peak current for screen-printed electrodes (μA); SQ = sum of squares; DF = degree of freedom; MQ = mean square; F_{calc} = Fisher distribution (calculated); F_{crit} = Fisher distribution (tabulated with a 5% of probability); A and B = electrodes that do not share a letter significantly differ.

Table S4. RSD of relative peak currents for 5.0 µmol L^{-1} of SFD in the presence of concomitant species at three different concentration levels (0.5, 5.0, and 50 µmol L^{-1}).

**Specie**	**Concentration (µmol L^{-1})**		
	0.50	5.0	50
	**RSD (%)**		
AA	+6.06	-1.50	-0.14
CA	-2.15	-3.08	+1.79
Ca^{2+}	-3.34	-2.83	+3.14
CAS	-6.91	-0.63	+6.70
GLU	+2.58	-5.59	+6.08
LAC	-3.44	-1.13	-3.52
Na^{+}	+3.52	+2.03	+1.35
SMX	-2.81	-7.55	-2.90
UR	-4.23	+1.82	+2.44

RSD = Relative standard deviation (%); AA = Ascorbic acid; CA = Citric acid; Ca^{2+} = Calcium ions; CAS = Casein; GLU = Glucose; LAC = Lactose; Na^{+} = Sodium ions; SMX = Sulfamethoxazole; UR = Urea.

References

(1) Oliveira, G. A.; Gevaerd, A.; Mangrich, A. S.; Marcolino-Junior, L. H.; Bergamini, M. F. Biochar Obtained from Spent Coffee Grounds: Evaluation of Adsorption Properties and Its Application in a Voltammetric Sensor for Lead (II) Ions. *Microchem. J.* **2021**, *165* (September 2020). https://doi.org/10.1016/j.microc.2021.106114.

(2) Möller, A.; Scholz, F. Advantages and Limitations of Combining Separation Techniques with Voltammetry. *Fresenius. J. Anal. Chem.* **1996**, *356* (3–4), 160–168. https://doi.org/10.1007/s0021663560160.

(3) Kalinke, C.; Zanicoski-Moscardi, A. P.; de Oliveira, P. R.; Mangrich, A. S.; Marcolino-Junior, L. H.; Bergamini, M. F. Simple and Low-Cost Sensor Based on Activated Biochar for the Stripping Voltammetric Detection of Caffeic Acid. *Microchem. J.* **2020**, *159* (July), 105380. https://doi.org/10.1016/j.microc.2020.105380.

(4) Cancelliere, R.; Di Tinno, A.; Di Lellis, A. M.; Tedeschi, Y.; Bellucci, S.; Carbone, K.; Signori, E.; Contini, G.; Micheli, L. An Inverse-Designed Electrochemical Platform for Analytical Applications. *Electrochem. commun.* **2020**, *121*, 106862. https://doi.org/10.1016/j.elecom.2020.106862.

(5) Ferreira, P. A.; Backes, R.; Martins, C. A.; de Carvalho, C. T.; da Silva, R. A. B. Biochar: A Low-Cost Electrode Modifier for Electrocatalytic, Sensitive and Selective Detection of Similar Organic Compounds. *Electroanalysis* **2018**, *30* (10), 2233–2236. https://doi.org/10.1002/elan.201800430.

(6) Valenga, M. G. P.; Martins, G.; Martins, T. A. C.; Didek, L. K.; Gevaerd, A.; Marcolino-Junior, L. H.; Bergamini, M. F. Biochar: An Environmentally Friendly Platform for Construction of a SARS-CoV-2 Electrochemical Immunosensor. *Sci. Total Environ.* **2023**, *858* (November 2022). https://doi.org/10.1016/j.scitotenv.2022.159797.
